# The Method of Types for the AWGN Channel †

**DOI:** 10.3390/e27060621

**Published:** 2025-06-11

**Authors:** Sergey Tridenski, Anelia Somekh-Baruch

**Affiliations:** Faculty of Engineering, Bar-Ilan University, Ramat Gan 5290002, Israel

**Keywords:** error exponents, method of types, Gaussian channel, reliability function, channel coding, correct-decoding

## Abstract

For the discrete-time AWGN channel with a power constraint, we give an alternative derivation for the sphere-packing upper bound on the optimal block error exponent and an alternative derivation for the analogous lower bound on the optimal correct-decoding exponent. The derivations use the method of types with finite alphabets of sizes depending on the block length *n* and with the number of types sub-exponential in *n*.

## 1. Introduction

We study reliability of the discrete-time additive white Gaussian noise (AWGN) channel with a power constraint imposed on blocks of its inputs. Consider the capacity of this channel, found by Shannon:

(1) $$C\;=\;\frac{1}{2}\;\log_{2}\left(1+s^{2}/\sigma^{2}\right),$$

where $\sigma^{2}$ is the channel noise variance and $s^{2}$ is the power constraint. This capacity corresponds to the maximum of the mutual information $I(\;p_{X},\;w)$ over $p_{X}$, under the power constraint on $p_{X}$, where *w* stands for the channel transition probability density function (PDF) and $p_{X}$ is the channel input PDF. Let us briefly recall the technicalities [1] of how expression (1) is obtained from the mutual information:

(2) maxpX:E[X2]≤s2I(pX,w)=maxpX:E[X2]≤s2Dw∥p^Y|pX−DpY∥p^Y=maxpX:E[X2]≤s212log(21+s2σ2+EX2−s22ln(2)(s2+σ2)−Dp(Y∥p^(Y︸≤0.

Here ${\hat{p}}_{Y}\left(y\right)≜\frac{1}{\sqrt{2\pi (s^{2}\;+\;\sigma^{2})}}e^{-\frac{y^{2}}{2(s^{2}\;+\;\sigma^{2})}}$ and $p_{Y}\left(y\right)\equiv \int_{R}p_{X}\left(x\right)w\left(y\;|\;x\right)dx$, the operator E[·] denotes the expectation, and *D* is the Kullback–Leibler divergence between two probability densities. The maximum in (2) is attained by the Gaussian PDF with zero mean and variance $s^{2}$, which simultaneously gives $E\left[X^{2}\right]=s^{2}$ and brings the divergence Dp(Y∥p^(Y to zero, which is its lowest possible value [1] [Equation (8.57)]. In this paper we consider the optimal exponents in the block error/correct-decoding probability of the AWGN channel. We propose explanations, similar to (2), both for Shannon’s sphere-packing converse bound on the optimal error exponent [2] [Equations (3), (4) and (11)] (see also [3] [Equation (47)] with [4] [Equations (7.5.34) and (7.5.32)]) and for Oohama’s converse bound on the optimal correct-decoding exponent [5] [Equation (4)].

In the case of discrete memoryless channels, the mutual information enters into the expressions for correct-decoding and error exponents through the method of types [6,7]. For the moment, without any particular meaning attached to it, let us rewrite the sphere-packing constant-composition exponent [6] [Equation (5.19)] with the PDF’s:

(3) minpY|X:I(p^(X,pY|X)≤RDp(Y|X∥w|p^(X,

where ${\hat{p}}_{X}$ denotes the Gaussian density with variance $s^{2}$, which maximizes (2), and R>0 is the information rate. When ${\hat{p}}_{X}$ is Gaussian, the minimum (3) allows an explicit solution by the method of Lagrange multipliers. The minimizing solution $p_{Y|X}^{*}$ of (3) is Gaussian [8] [Equation (65)], Ref. [9] [Equation (327)], and we obtain that (3) is the same as Shannon’s converse bound on the error exponent [2] [Equations (3), (4) and (11)], Refs. [8,9] in the limit of a large block length:

(4) $$\begin{array}{cc} E_{sp}\left(R\right) & =\;\left[\;\frac{1}{2}A^{2}\;-\;\frac{1}{2}AG\cos\theta \;-\;\ln\left(G\sin\theta \right)\;\right]\;\log_{2}\;e,\\ G & =\;\frac{1}{2}\left(A\cos\theta \;+\;\sqrt{A^{2}\cos^{2}\theta +4}\right),\;\;\;\sin\theta \;=\;2^{-\min\;\{R,\;C\}},\;\;\;A\;=\;s/\sigma . \end{array}$$

Then it turns out that $p_{Y|X}^{*}$ of (3) and the *y*-marginal PDF of the product ${\hat{p}}_{X}p_{Y|X}^{*}$ [8] [Equation (63)] play the same roles in the derivation of the converse bound as *w* and ${\hat{p}}_{Y}$, respectively, in the maximization (2).

In this paper, in order to derive expressions similar to (3), we extend the method of types [1] [Chapter 11.1], Ref. [10] to include countable alphabets consisting of uniformly spaced real numbers, with the help of power constraints on types. The countable alphabets depend on the block length *n* and the number of types satisfying the power constraints is kept sub-exponential in *n*. The latter idea is inspired by a different subject—of “runs” in a binary sequence. If we treat every “run” of ones or zeros in a binary sequence as a separate symbol from the countable alphabet of run-lengths, then the number of different empirical distributions of such symbols in a binary sequence of length *n* is equivalent to the number of different partitions of the integer *n* into the sum of positive integers, which is $∼e^{c\sqrt{n}}$ [11] [Equation (5.1.2)]. Thus, it is sub-exponential, and the method of types can be extended to that case. In our present case, however, the types are empirical distributions of uniformly quantized real numbers in quantized versions of real channel input and output vectors of length *n*. The quantized versions serve only for classification of channel input and output vectors and not for the communication itself. The uniform quantization step is different for the quantized versions of channel inputs and outputs, and in both cases it is chosen to be a decreasing function of *n*.

Via expressions similar to (2), the proposed derivations demonstrate that, in order to achieve the converse bounds on the correct-decoding and error exponents, it is necessary for the types of the quantized versions of codewords to converge to the Gaussian distribution in the characteristic function (CF), or, equivalently, in cumulative distribution function (CDF).

The contributions of the current paper are twofold: we successfully apply the method of types to derive converse bounds on both the error exponent [12] and the correct-decoding exponent [13] of the AWGN channel. This underscores the advantage of the method of types.

In Section 2 and Section 3, we describe the communication system and introduce other definitions. In Section 4 we present the main results of the paper, which consist of two theorems and a proposition. Section 5 provides an extension to the method of types. The results of Section 5 are then applied in all the sections that follow. In Section 6 we prove a converse lemma that is then used for derivation of both the correct-decoding and error exponents in Section 7 and Section 8, respectively. Section 9 connects between the PDFs and types.

### Notation

Countable alphabets consisting of real numbers are denoted by $X_{n}$, $Y_{n}$. The set of types with denominator *n* over $X_{n}$ is denoted by $P_{n}\left(X_{n}\right)$. Capital ‘P’ denotes probability mass functions, which are types $P_{X}$, $P_{Y}$, $P_{XY}$, $P_{Y|X}$. The type class and the support of a type $P_{X}$ are denoted by $T(P_{X})$ and $S(P_{X})$, respectively. The expectation with respect to a probability distribution $P_{X}$ is denoted by $E_{P_{X}}\left[\;\cdot \;\right]$. Small ‘*p*’ denotes probability density functions $p_{X}$, $p_{Y}$, $p_{XY}$, $p_{Y|X}$. Thin letters *x*, *y* represent real values, while thick letters x, y represent real vectors. Capital letters *X*, *Y* represent random variables; boldface Y represents a random vector of length *n*. The conditional type class of $P_{X|\;Y}$ given y is denoted by $T(P_{X|\;Y}\;|\;y)$. The quantized versions of variables are denoted by a superscript ‘*q*’: $x_{k}^{q}$, $x^{q}$, $Y^{q}$. Small *w* stands for a conditional PDF, and $W_{n}$ stands for a discrete positive measure which does not necessarily add up to 1. All information-theoretic quantities such as joint and conditional entropies $H(P_{XY})$, H(Y|X), the mutual information $I(P_{XY})$, $I\left(P_{X},P_{Y|X}\right)$, $I\left(P_{X},\;p_{Y|X}\right)$, the Kullback–Leibler divergence $D\left(P_{Y|X}\;∥\;W_{n}\;|\;P_{X}\right)$, $D\left(\;p_{Y|X}\;∥\;\;w\;\;|\;P_{X}\right)$, and the information rate *R* are defined with respect to the logarithm to a base b>1, denoted as $\log_{b}\left(\cdot \right)$. It is assumed that $0\;\log_{b}\left(0\right)=0$. The natural logarithm is denoted as ln. The cardinality of a discrete set is denoted by |·|, while the volume of a continuous region is denoted by vol(·). The complementary set of a set *A* is denoted by $A^{c}$. Logical “or” and “and” are represented by the symbols ∨ and ∧, respectively. Gaussian distributions are denoted by N, while Unif(·) stands for the discrete uniform distribution. In the Appendix B, $p_{XY}^{q}$ represents the rounded down version of the PDF $p_{XY}$.

## 2. Communication System

We consider communication over the time-discrete additive white Gaussian noise channel with real channel inputs x∈R and channel outputs y∈R and a transition probability density

$$w\left(y\;|\;x\right)\;\;≜\;\;\frac{1}{\sigma \sqrt{2\pi }}e^{-\frac{{\left(y\;-\;x\right)}^{2}}{2\sigma^{2}}}.$$

Communication is performed by blocks of *n* channel inputs. Let R>0 denote a nominal information rate. Each block is used for transmission of one out of *M* messages, where $M=M\left(n,R\right)≜\left\lfloor b^{\;nR}\right\rfloor$, for some logarithm base b>1. The encoder is a deterministic function $f:\left\{1,2,\ldots ,M\right\}\to R^{n}$, which converts a message into a transmitted block, such that

$$f\left(m\right)\;=\;x\left(m\right)\;=\;\left(x_{1}\left(m\right),\;x_{2}\left(m\right),\ldots ,\;x_{n}\left(m\right)\right),\;\;\;\;\;\;\;\;\;m=1,2,\ldots ,\;M,$$

where $x_{k}\left(m\right)\in R$, for all k=1,2,…,n. The set of all the codewords x(m), m=1,2,…,M, constitutes a codebook C. Each codeword x(m) in C satisfies the power constraint:

(5) $$\frac{1}{n}\sum_{k\;=\;1}^{n}x_{k}^{2}\left(m\right)\;\leq \;s^{2},\;\;\;\;\;\;\;\;\;m=1,2,\ldots ,\;M.$$

The decoder is another deterministic function $g:R^{n}\to \left\{0,1,2,\ldots ,\;M\right\}$, which converts the received block of *n* channel outputs $y\in R^{n}$ into an estimated message or, possibly, to a special error symbol ‘0’:

(6) $$g\left(y\right)\;\;=\;\;\{\begin{array}{cc} 0, & \;\;\;y\in \overset{\;M}{\underset{\;m\;=\;1}{⋂}}D_{m}^{c},\\ m, & \;\;\;y\in D_{m},\;\;\;m\in \left\{1,2,\ldots ,\;M\right\}, \end{array}$$

where each set $D_{m}\subseteq R^{n}$ is either an open region or the empty set, and the regions are disjoint: $D_{m}\cap \;D_{m^{\prime }}=⌀$ for $m\neq m^{\prime }$. Observe that the maximum-likelihood decoder with open decision regions $D_{m}^{*}\;$, defined for m=1,2,…,M as

$$D_{m}^{*}\;\;=\;\;R^{n}\setminus \underset{m^{\prime }:\;\;\left(m^{\prime }\;<\;m\right)\;\vee \;\left(\;m^{\prime }\;>\;m\;\;\wedge \;\;x\left(m^{\prime }\right)\;\neq \;x\left(m\right)\;\right)}{⋃}\left\{y:\;∥y-x\left(m^{\prime }\right)∥\;\leq \;∥y-x\left(m\right)∥\right\},$$

is a special case of (6). Note that the formal definition of $D_{m}^{*}$ includes the undesirable possibility of $x\left(m^{\prime }\right)=x\left(m\right)$ for $m^{\prime }\neq m$.

## 3. Definitions

For each *n*, we define two discrete countable alphabets $X_{n}$ and $Y_{n}$ as one-dimensional lattices:

α,β,γ∈(0,1),α+β+γ=1,

(7) $$\Delta_{\alpha ,n}\;≜\;1/n^{\alpha },\;\;\;\Delta_{\beta ,n}\;≜\;1/n^{\beta },\;\;\;\Delta_{\gamma ,n}\;≜\;1/n^{\gamma },$$

(8) $$\Delta_{\alpha ,n}\cdot \Delta_{\beta ,n}\cdot \Delta_{\gamma ,n}\;\;=\;\;1/n,$$

(9) $$X_{n}\;\;≜\;\;\underset{i\;\in \;Z}{⋃}\left\{i\Delta_{\alpha ,n}\right\},\;\;\;\;\;\;Y_{n}\;\;≜\;\;\underset{i\;\in \;Z}{⋃}\left\{i\Delta_{\beta ,n}\right\}.$$

For each *n*, we define also a discrete positive measure (not necessarily a distribution), which will approximate the channel *w*:

(10) $$W_{n}\left(y\;|\;x\right)\;\;≜\;\;w\left(y\;|\;x\right)\cdot \Delta_{\beta ,n},\;\;\;\forall x\in X_{n},\;\forall y\in Y_{n}.$$

Denoting by $C^{0}\left(A\right)$ a class of functions $f:R\to R_{\;\geq \;0}$ continuous on an open subset A⊆R, we define

(11) $$\begin{array}{cc} F_{n}\; & ≜\;\left\{f:R\to R_{\;\geq \;0}\;\;\;|\;\;\;\underset{y\;\in \;R}{\sup}f\left(y\right)<+\infty ;\;\;\;f\in C^{0}\left(R\setminus \{Y_{n}+\Delta_{\beta ,n}/2\}\right);\;\;\int_{R}f\left(y\right)dy=1\right\}. \end{array}$$

The set (11), defined for a given *n*, will be used only in the derivation of the correct-decoding exponent, while the following set of Lipschitz continuous functions will be used only in the derivation of the error exponent:

(12) $$\begin{array}{cc} L\; & ≜\;\left\{f:R\to R_{\;\geq \;0}\;\;|\;\;|f\left(y_{1}\right)-f\left(y_{2}\right)\left|\leq K\right|y_{1}-y_{2}|,\;\forall y_{1},y_{2}\;;\;\int_{R}f\left(y\right)dy=1\right\},\;\;K≜\frac{1}{\sigma^{2}\sqrt{2\pi e}}. \end{array}$$

Note that L is a convex set and also each function f∈L is bounded and cannot exceed $\sqrt{K}$.

With a parameter ρ∈(−1,+∞), we define the following Gaussian probability density functions [8,9]:

(13) $$\begin{array}{cc} p_{Y|X}^{\left(\rho \right)}\left(y\;|\;x\right)\;\; & ≜\;\;\frac{1}{\sigma_{Y|X}\left(\rho \right)\sqrt{2\pi }}\exp\left\{-\frac{{\left(y\;-\;k_{\rho }\;\cdot \;x\right)}^{2}}{2\sigma_{Y|X}^{2}\left(\rho \right)}\right\}, \end{array}$$

(14) $$\begin{array}{cc} k_{\rho }\;\; & ≜\;\;\frac{\mathrm{SNR}\;-\;\rho \;-\;1\;+\;\sqrt{{\left(\mathrm{SNR}\;-\;\rho \;-\;1\right)}^{2}\;+\;4\;\cdot \;\mathrm{SNR}}}{2\;\cdot \;\mathrm{SNR}},\;\;\;\;\;\;\mathrm{SNR}\;≜\;s^{2}/\sigma^{2}, \end{array}$$

(15) $$\begin{array}{cc} \sigma_{Y|X}^{2}\left(\rho \right)\;\; & ≜\;\;\left(1+\rho \right)k_{\rho }\;\sigma^{2}, \end{array}$$

(16) $$\begin{array}{cc} {\hat{p}}_{Y}^{\;(\rho )}\left(y\right)\;\; & ≜\;\;\frac{1}{\sigma_{Y}\left(\rho \right)\sqrt{2\pi }}\exp\left\{-\frac{y^{2}}{2\sigma_{Y}^{2}\left(\rho \right)}\right\}, \end{array}$$

(17) $$\begin{array}{cc} \sigma_{Y}^{2}\left(\rho \right)\;\; & ≜\;\;\sigma^{2}\;+\;k_{\rho }\;s^{2}, \end{array}$$

(18) $$\begin{array}{cc} {\hat{p}}_{X}\left(x\right)\;\; & ≜\;\;\frac{1}{s\sqrt{2\pi }}\exp\left\{-\frac{x^{2}}{2s^{2}}\right\}. \end{array}$$

The first property of the following lemma shows that ${\hat{p}}_{Y}^{\;(\rho )}$ is the *y*-marginal PDF of the product $\;{\hat{p}}_{X}p_{Y|X}^{\left(\rho \right)}$.

**Lemma** **1** (Properties of (13)–(18))**.** *The following properties hold:*

(19) $$\begin{array}{cc} \sigma_{Y}^{2}\left(\rho \right)\;\; & =\;\;\sigma_{Y|X}^{2}\left(\rho \right)\;+\;k_{\rho }^{2}\;s^{2}, \end{array}$$

(20) $$\begin{array}{cc} \frac{1+\rho }{\sigma_{Y|X}^{2}\left(\rho \right)}\;\; & =\;\;\frac{\rho }{\sigma_{Y}^{2}\left(\rho \right)}\;+\;\frac{1}{\sigma^{2}}, \end{array}$$

(21) $$\begin{array}{cc} \sigma_{Y|X}^{2}\left(\rho \right)\;\; & =\;\;\sigma^{2}\;+\;k_{\rho }\left(1-k_{\rho }\right)s^{2}, \end{array}$$

(22) $$\begin{array}{cc} 1\; & \geq \;k_{\rho }\;>\;0,\;\;\;\;\;\;\;\;\;\;\;\;\;\;\;\;\;\rho \geq 0, \end{array}$$

(23) $$\begin{array}{cc} \frac{1}{2}\left[1\;+\;\sqrt{1\;+\;4\sigma^{2}s^{-2}}\;\right]\; & \geq \;k_{\rho }\;\geq \;1,\;\;\;\;\;\;-1\leq \rho \leq 0, \end{array}$$

(24) $$\begin{array}{cc} p_{Y|X}^{\left(\rho \right)}\left(\;\cdot \;\;|\;x\right)\; & \in \;L,\;\;\;\;\;\;\forall \rho \geq 0,\;\forall x\in R, \end{array}$$

*and for any two jointly distributed random variables (X,Y), such that $E\left[X^{2}\right]=\sigma_{X}^{2}\;\leq \;s^{2}+\epsilon$, ϵ>0, and $Y\;|\;X=x\;∼\;N\left(k_{\rho }\;x,\;\sigma_{Y|X}^{2}\left(\rho \right)\right)$, it holds that*

(25) $$\begin{array}{cc} E\left[{\left(Y-X\right)}^{2}\right]\;\; & =\;\;\sigma^{2}\;+\;\left(1-k_{\rho }\right)s^{2}\;+\;{\left(1-k_{\rho }\right)}^{2}\left(\sigma_{X}^{2}-s^{2}\right) \end{array}$$

(26) $$\begin{array}{cc} \; & \leq \;\;\{\begin{array}{cc} \sigma^{2}\;+\;s^{2}\;+\;\epsilon , & \;\;\;\;\;\;\rho \geq 0,\\ \sigma^{2}\;+\;\epsilon \sigma^{2}s^{-2}, & \;\;\;\;\;\;-1<\rho \leq 0. \end{array} \end{array}$$

Here (17) combined with (14) corresponds to [8] [Equation (64)], (15) and (20) can be found in [8] [Equation (65)], while (14), (21) correspond respectively to [9] [Equations (302) and (328)].

**Proof** **of Lemma 1.** The first property (19) can be verified using (14), (15), (17). Then (20) can be obtained from (15), (17), (19). Property (21) follows by (17) and (19). It can be verified from (14) that $k_{\rho }$ is a positive monotonically decreasing function of ρ, such that $k_{0}=1$. Then we get (22) and (23). From (21) and (22) we see that $\sigma_{Y|X}^{2}\left(\rho \right)\geq \sigma^{2}$ for all ρ≥0, which gives (24). Equality (25) can be obtained using (21). Then, using (22) and (23), we obtain (26). □

The following expressions will describe our results for the error and correct-decoding exponents:

(27) $$\begin{array}{cc} E_{e}\left(R\right)\;\; & ≜\;\;\;\;\underset{\rho \;\geq \;0}{\sup}\;\;\left\{D\left(\;p_{Y|X}^{\left(\rho \right)}\;∥\;\;w\;\;|\;\;{\hat{p}}_{X}\right)\;+\;\rho \left[I\left(\;{\hat{p}}_{X},\;p_{Y|X}^{\left(\rho \right)}\right)\;-\;R\right]\right\}, \end{array}$$

(28) $$\begin{array}{cc} E_{c}\left(R\right)\;\; & ≜\;\;\underset{-1\;<\;\rho \;\leq \;0}{\sup}\left\{D\left(\;p_{Y|X}^{\left(\rho \right)}\;∥\;\;w\;\;|\;\;{\hat{p}}_{X}\right)\;+\;\rho \left[I\left(\;{\hat{p}}_{X},\;p_{Y|X}^{\left(\rho \right)}\right)\;-\;R\right]\right\}. \end{array}$$

The following identity can be obtained using (13), (15), (16), (18) and (20):

$$D\left(\;p_{Y|X}^{\left(\rho \right)}\;∥\;\;w\;\;|\;\;{\hat{p}}_{X}\right)\;+\;\rho I\left(\;{\hat{p}}_{X},\;p_{Y|X}^{\left(\rho \right)}\right)\;\;\equiv \;\;c_{0}\left(\rho \right)\;+\;c_{1}\left(\rho \right)s^{2},$$

(29) $$c_{0}\left(\rho \right)\;\;≜\;\;\frac{1}{\ln b}\ln\left(\frac{\sigma \cdot \sigma_{Y}^{\rho }\left(\rho \right)}{\sigma_{Y|X}^{1\;+\;\rho }\left(\rho \right)}\right),\;\;\;\;\;\;\;\;\;c_{1}\left(\rho \right)\;\;≜\;\;\frac{1-k_{\rho }}{2\sigma^{2}\ln b}.$$

We note also that $c_{0}\left(\rho \right)\to 0$, as ρ→+∞, which can be verified using the properties (15), (17) and (21). It can be verified that the expression inside the supremum of (27) is equivalent to the expression for the Gaussian random-coding error exponent of Gallager before the maximization over ρ [4] [Equations (7.4.24) and (7.4.28)]. Therefore, with the supremum over ρ≥0, the expression (27) coincides with the converse sphere-packing bound of Shannon (4).

## 4. Main Results

In this section we present two theorems and a proposition. The two theorems give converse bounds on the optimal error exponent and correct-decoding exponent of the AWGN, respectively. The bounds are asymptotic in the limit of a large block length *n*, and are given by the expressions (27) and (28), accordingly. The proofs, leading to these expressions, are also presented in this section, while some of their technical details are encapsulated in the form of lemmas that are taken care of by the rest of the sections in this paper. In the course of the two proofs leading to (27) and (28) we obtain expressions analogous to (2), which, in exactly the same manner as the maximization of (2), allow us to draw conclusions about the asymptotically optimal codeword types, achieving the converse bounds. This is made precise in the remark below, after the proof of the second theorem. The section is concluded with the proposition that brings together the bounds of the two theorems in a parametric form.

The proof of the first theorem relies on Lemmas 13 and 19, which appear in Section 7 and Section 9, respectively.

**Theorem** **1** (Error exponent)**.** *Let $J∼Unif\;\left(\{1,2,\ldots ,M\}\right)$ be a random variable, independent of the channel noise, and let x(J)→Y be the random channel-input and channel-output vectors, respectively. Then*

$$\begin{array}{cc} \underset{\begin{array}{c} n\;\to \;\infty \end{array}}{lim sup}\;\underset{C}{\sup}\;\underset{g}{\sup}\;\left\{-\frac{1}{n}\;\log_{b}\mathrm{Pr}\left\{g(Y)\neq J\right\}\right\}\;\; & \leq \;\;E_{e}\left(R\right), \end{array}$$

*where $E_{e}\left(R\right)$ is defined by (27), decoder functions g are defined by (6), and codebooks C satisfy (5).*

**Proof.**  Starting from Lemma 13, we can write the following sequence of inequalities:

(30) $$\underset{\begin{array}{c} n\;\to \;\infty \end{array}}{lim sup}\;\;\;\underset{C}{\sup}\;\;\underset{g}{\sup}\;\;\left\{-\frac{1}{n}\;\log_{b}\mathrm{Pr}\left\{g(Y)\neq J\right\}\right\}$$

(31) $$\overset{a}{\leq }\underset{n\;\to \;\infty }{lim sup}\max_{\begin{array}{c} P_{X}:\\ P_{X}\;\in \;P_{n}\left(X_{n}\right),\\ E\left[X^{2}\right]\;\leq \;s^{2}\;+\;\epsilon \end{array}}\;\;\;\;\;\;\;\;\min_{\begin{array}{c} P_{Y|X}:\\ P_{XY}\;\in \;P_{n}\left(X_{n}\;\times \;Y_{n}\right),\\ E\left[{\left(Y-X\right)}^{2}\right]\;\leq \;\sigma^{2}\;+\;s^{2}\;+\;2\epsilon ,\\ I(P_{X},\;P_{Y|X})\;\leq \;R\;-\;\epsilon \end{array}}D\left(P_{Y|X}\;∥\;W_{n}\;|\;P_{X}\right)$$

(32) $$\overset{b}{\leq }\underset{n\;\to \;\infty }{lim sup}\;\max_{\begin{array}{c} P_{X}:\\ P_{X}\;\in \;P_{n}\left(X_{n}\right),\\ E\left[X^{2}\right]\;\leq \;s^{2}\;+\;\epsilon \end{array}}\;\;\;\;\;\;\;\;\underset{\begin{array}{c} p_{Y|X}:\\ p_{Y|X}\left(\;\cdot \;|\;x\right)\;\in \;L,\;\forall x,\\ E\left[{\left(Y-X\right)}^{2}\right]\;\leq \;\sigma^{2}\;+\;s^{2}\;+\;\epsilon ,\\ I(P_{X},\;\;p_{Y|X})\;\leq \;R\;-\;2\epsilon \end{array}}{\inf}D\left(p_{Y|X}\;∥\;\;w\;\;|\;P_{X}\right)$$

(33) $$\overset{c}{=}\underset{n\;\to \;\infty }{lim sup}\;\max_{\begin{array}{c} P_{X}:\\ P_{X}\;\in \;P_{n}\left(X_{n}\right),\\ E\left[X^{2}\right]\;\leq \;s^{2}\;+\;\epsilon \end{array}}\;\underset{\begin{array}{c} \rho \;\geq \;0 \end{array}}{\sup}\underset{\begin{array}{c} p_{Y|X}:\\ p_{Y|X}\left(\;\cdot \;|\;x\right)\;\in \;L,\;\forall x,\\ E\left[{\left(Y-X\right)}^{2}\right]\;\leq \;\sigma^{2}\;+\;s^{2}\;+\;\epsilon \end{array}}{\inf}\left\{D\left(\;p_{Y|X}\;∥\;\;w\;\;|\;P_{X}\right)\;+\;\rho \left[I\left(P_{X},\;p_{Y|X}\right)-R+2\epsilon \right]\right\}$$

$$\overset{d}{\leq }\underset{n\;\to \;\infty }{lim sup}\;\max_{\begin{array}{c} P_{X}:\\ P_{X}\;\in \;P_{n}\left(X_{n}\right),\\ E\left[X^{2}\right]\;\leq \;s^{2}\;+\;\epsilon \end{array}}\underset{\rho \;\geq \;0}{\sup}\;\;\;\;\;\;\;\;\;\;\;\;\;\;\;\;\;\;\;\;\;\;\;\;\;\;\;\;\;\left\{D\left(p_{Y|X}^{\left(\rho \right)}\;∥\;\;w\;\;|\;P_{X}\right)\;+\;\rho \left[I\left(P_{X},\;p_{Y|X}^{\left(\rho \right)}\right)-R+2\epsilon \right]\right\}$$

(34) $$\overset{e}{\equiv }\underset{n\;\to \;\infty }{lim sup}\;\max_{\begin{array}{c} P_{X}:\\ P_{X}\;\in \;P_{n}\left(X_{n}\right),\\ E\left[X^{2}\right]\;\leq \;s^{2}\;+\;\epsilon \end{array}}\;\underset{\rho \;\geq \;0}{\sup}\;\;\;\;\;\;\;\;\;\;\;\;\;\;\left\{c_{0}\left(\rho \right)\;+\;c_{1}\left(\rho \right)\;E\left[X^{2}\right]\;+\;\rho \left[-D\left(\;p_{Y}^{\;(\rho )}\;∥\;{\hat{p}}_{Y}^{\left(\rho \right)}\right)-R+2\epsilon \right]\right\}$$

(35) $$\overset{f}{\leq }\;\;\;\;\;\;\;\;\;\;\;\;\;\;\;\;\;\;\;\;\;\;\;\;\;\;\;\;\;\;\;\;\;\underset{\rho \;\geq \;0}{\sup}\;\;\;\;\;\;\;\;\;\;\;\;\;\;\;\;\left\{c_{0}\left(\rho \right)\;+\;c_{1}\left(\rho \right)\left(s^{2}+\epsilon \right)\;-\;\rho \left(R-2\epsilon \right)\right\},$$

where

(*a*) holds for any ϵ>0 by Lemma 13 with $c_{XY}=\sigma^{2}+s^{2}+2\epsilon$. Note also that $D\left(P_{Y|X}\;∥\;W_{n}\;|\;P_{X}\right)$ in (31) denotes the Kullback–Leibler divergence between the probability distribution $P_{Y|X}$ and the positive measure $W_{n}$ defined in (10), which is not a probability distribution but only approximates the channel *w*.

(*b*) follows by Lemma 19 for the alphabet parameters $\alpha \in \left(0,\frac{1}{4}\right)$ and $\frac{1}{3}+\frac{2}{3}\alpha <\beta <\frac{2}{3}-\frac{2}{3}\alpha$.

(*c*) holds for all R>0 with the possible exception of the *single point* on *R*-axis where (32) may transition between a finite value and +∞. To verify the equality, let us compare the infimum in (32) and the supremum over ρ≥0 in (33) as functions of R∈R, for a given $P_{X}$. First, it can be verified that the supremum of (33) is the closure of the lower convex envelope of the infimum of (32). Second, it can be checked by the definition of convexity, that the infimum of (32) itself is a convex (∪) function of *R*. Then they coincide for all values of *R*, except possibly for the single point where they both jump to +∞. This property carries over to the external ‘lim sup max’ as well.

(*d*) follows because by (24) and (26) function $p_{Y|X}^{\left(\rho \right)}$ satisfies the conditions under the infimum of (33).

(*e*) holds as equality inside the supremum over ρ≥0, separately for each ρ. In (34) by $p_{Y}^{\;(\rho )}$ we denote the corresponding marginal PDF of the product $P_{X}p_{Y|X}^{\left(\rho \right)}$ and use the definitions (29). Then (*e*) follows by the definitions of $p_{Y|X}^{\left(\rho \right)}$ and ${\hat{p}}_{Y}^{\;(\rho )}$ in (13) and (16), and by their properties (15) and (20).

(*f*) follows by the non-negativity of the divergence, and by the condition under the maximum of (34), since $c_{1}\left(\rho \right)\geq 0$ for ρ≥0.

In conclusion, according to (*c*) we obtain that the inequality between (30) and (35), as functions of *R*, holds for all R>0, except possibly for the single point R=2ϵ, where the jump to +∞ in (35) occurs. Therefore, taking the limit as ϵ→0, we obtain that (30) is upper-bounded for all R>0 by

$$\underset{\rho \;\geq \;0}{\sup}\;\left\{c_{0}\left(\rho \right)\;+\;c_{1}\left(\rho \right)s^{2}\;-\;\rho R\right\},$$

which is the same as (27). □

The second theorem relies on Lemmas 17 and 20, which appear in Section 8 and Section 9, respectively.

**Theorem** **2** (Correct-decoding exponent)**.** *Let $J∼Unif\;\left(\{1,2,\ldots ,\;M\}\right)$ be a random variable, independent of the channel noise, and let x(J)→Y be the random channel-input and channel-output vectors, respectively. Then*

$$\underset{n\;\to \;\infty }{lim inf}\;\underset{C}{\inf}\;\underset{g}{\inf}\;\left\{-\frac{1}{n}\log_{b}\mathrm{Pr}\left\{g(Y)=J\right\}\right\}\;\;\geq \;\;E_{c}\left(R\right),$$

*where $E_{c}\left(R\right)$ is defined by (28), decoder functions g are defined by (6), and codebooks C satisfy (5).*

**Proof.**  Starting from Lemma 17, for each R>0 we can choose a different parameter $\tilde{\sigma }=\tilde{\sigma }\left(R\right)\geq \sigma$, such that there is equality $E\left(\tilde{\sigma }\left(R\right)\right)=E_{c}\left(R\right)$ between (74) and (28). Then by (80) we obtain

$$\underset{n\;\to \;\infty }{lim inf}\;\underset{C}{\inf}\;\underset{g}{\inf}\;\left\{-\frac{1}{n}\log_{b}\mathrm{Pr}\left\{g(Y)=J\right\}\right\}\;\;\geq \;\;\min\left\{\underset{n\;\to \;\infty }{lim inf}\;E_{n}\left(R,\;\tilde{\sigma }\left(R\right),\;\tilde{\epsilon },\;\epsilon \right),\;\;E_{c}\left(R\right)\right\}.$$

With the choice $2\tilde{\epsilon }=\epsilon \sigma^{2}s^{-2}$, the first term in the minimum can be lower-bounded as follows:

$$\begin{array}{ccccc} \; & \underset{n\;\to \;\infty }{lim inf} & \min_{\begin{array}{c} P_{X}:\\ P_{X}\;\in \;P_{n}\left(X_{n}\right),\\ E\left[X^{2}\right]\;\leq \;s^{2}\;+\;\epsilon \end{array}} & \min_{\begin{array}{c} P_{Y|X}:\\ P_{XY}\;\in \;P_{n}\left(X_{n}\;\times \;Y_{n}\right),\\ E\left[{\left(Y-X\right)}^{2}\right]\;\leq \;{\tilde{\sigma }}^{2}\left(R\right)\;+\;\tilde{\epsilon } \end{array}} & \left\{D\left(P_{Y|X}\;∥\;W_{n}\;|\;P_{X}\right)\;+\;|\;R\;-\;I\left(P_{X},P_{Y|X}\right)\;{|}^{+}\right\}\\ \overset{a}{\geq } & \underset{n\;\to \;\infty }{lim inf} & \min_{\begin{array}{c} P_{X}:\\ P_{X}\;\in \;P_{n}\left(X_{n}\right),\\ E\left[X^{2}\right]\;\leq \;s^{2}\;+\;\epsilon \end{array}} & \underset{\begin{array}{c} p_{Y|X}:\\ p_{Y|X}\left(\;\cdot \;\;|\;x\right)\;\in \;F_{n},\;\forall x,\\ E\left[{\left(Y-X\right)}^{2}\right]\;\leq \;{\tilde{\sigma }}^{2}\left(R\right)\;+\;2\tilde{\epsilon } \end{array}}{\inf} & \left\{D\left(\;p_{Y|X}\;∥\;\;w\;\;|\;P_{X}\right)\;+\;|\;R\;-\;I\left(P_{X},\;p_{Y|X}\right)\;{|}^{+}\right\}\\ \overset{b}{\geq } & \underset{n\;\to \;\infty }{lim inf} & \min_{\begin{array}{c} P_{X}:\\ P_{X}\;\in \;P_{n}\left(X_{n}\right),\\ E\left[X^{2}\right]\;\leq \;s^{2}\;+\;\epsilon \end{array}} & \underset{\begin{array}{c} p_{Y|X}:\\ p_{Y|X}\left(\;\cdot \;|\;x\right)\;\in \;F_{n},\;\forall x,\\ E\left[{\left(Y-X\right)}^{2}\right]\;\leq \;{\tilde{\sigma }}^{2}\left(R\right)\;+\;2\tilde{\epsilon } \end{array}}{\inf} & \left\{D\left(\;p_{Y|X}\;∥\;\;w\;\;|\;P_{X}\right)\;-\;\rho \left[R\;-\;D\left(\;p_{Y|X}\;∥\;\;{\hat{p}}_{Y}^{\;(\rho )}\;|\;P_{X}\right)\right]\right\}\\ \overset{c}{\equiv } & \underset{n\;\to \;\infty }{lim inf} & \min_{\begin{array}{c} P_{X}:\\ P_{X}\;\in \;P_{n}\left(X_{n}\right),\\ E\left[X^{2}\right]\;\leq \;s^{2}\;+\;\epsilon \end{array}} & \underset{\begin{array}{c} p_{Y|X}:\\ p_{Y|X}\left(\;\cdot \;|\;x\right)\;\in \;F_{n},\;\forall x,\\ E\left[{\left(Y-X\right)}^{2}\right]\;\leq \;{\tilde{\sigma }}^{2}\left(R\right)\;+\;2\tilde{\epsilon } \end{array}}{\inf} & \left\{c_{0}\left(\rho \right)\;+\;c_{1}\left(\rho \right)\;E\left[X^{2}\right]\;-\;\rho R\;+\;\left(1+\rho \right)D\left(\;p_{Y|X}\;∥\;\;p_{Y|X}^{\left(\rho \right)}\;|\;P_{X}\right)\right\} \end{array}$$

(36) $$\overset{d}{=}\underset{\begin{array}{c} n\;\to \;\infty \end{array}}{lim inf}\min_{\begin{array}{c} P_{X}:\\ P_{X}\;\in \;P_{n}\left(X_{n}\right),\\ E\left[X^{2}\right]\;\leq \;s^{2}\;+\;\epsilon \end{array}}\;\;\;\;\;\;\;\;\;\;\;\;\;\;\;\;\;\;\;\;\;\;\;\;\;\;\;\;\;\;\;\;\;\left\{c_{0}\left(\rho \right)\;+\;c_{1}\left(\rho \right)\;E\left[X^{2}\right]\;-\;\rho R\right\}$$

(37) $$\overset{e}{\geq }\;\;\;\;\;\;\;\;\;\;\;\;\;\;\;\;\;\;\;\;\;\;\;\;\;\;\;\;\;\;\;\;\;\;\;\;\;\;\;\;\;\;\;\;\;\;\;\;\;\;\;\;\;\;\;\;\;\;\;\;\;\;\;\;c_{0}\left(\rho \right)\;+\;c_{1}\left(\rho \right)\left(s^{2}+\epsilon \right)\;-\;\rho R,$$

where:

(*a*) follows by Lemma 20 with $c_{XY}={\tilde{\sigma }}^{2}\left(R\right)+\tilde{\epsilon }$.

(*b*) holds for ρ∈(−1,0], because $|\;R\;-\;I\left(P_{X},\;p_{Y|X}\right)\;{|}^{+}\;\geq \;-\rho \left[\;R\;-\;I\left(P_{X},\;p_{Y|X}\right)\;\right]$ for any such ρ, and because $I\left(P_{X},\;p_{Y|X}\right)\leq D\left(\;p_{Y|X}\;∥\;\;{\hat{p}}_{Y}^{\;(\rho )}\;|\;P_{X}\right)$, where ${\hat{p}}_{Y}^{\;(\rho )}$ is the Gaussian PDF defined in (16).

(*c*) holds as an identity inside the infimum by the definitions (13), (16) and (29), and properties (15) and (20).

(*d*) holds if $2\tilde{\epsilon }\geq \epsilon \sigma^{2}s^{-2}$ and ρ∈(−1,0], because then by (26) and (11) the function $p_{Y|X}^{\left(\rho \right)}$ satisfies the conditions under the infimum and achieves the infimum.

(*e*) follows by the condition under the minimum of (36) since $c_{1}\left(\rho \right)\leq 0$ for ρ∈(−1,0].

In conclusion, since (37) is the lower bound for any ρ∈(−1,0] and $2\tilde{\epsilon }\geq \epsilon \sigma^{2}s^{-2}$, we obtain

$$\underset{\begin{array}{c} n\;\to \;\infty \end{array}}{lim inf}\;E_{n}\left(R,\;\tilde{\sigma }\left(R\right),\;\epsilon \sigma^{2}s^{-2}/2,\;\epsilon \right)\;\;\geq \;\;\underset{-1\;<\;\rho \;\leq \;0}{\sup}\left\{c_{0}\left(\rho \right)\;+\;c_{1}\left(\rho \right)\left(s^{2}+\epsilon \right)\;-\;\rho R\right\}\;\;\overset{\epsilon \;\to \;0}{⟶}\;\;E_{c}\left(R\right).$$

□

**Remark** **1.** *Observe that neither the inequality (f) in the proof of Theorem 1 nor the inequality (e) in the proof of Theorem 2 can be met with equality unless $E\left[X^{2}\right]\to s^{2}+\epsilon$. Furthermore, neither the inequality (f) in the proof of Theorem 1 nor the inequality (b) in the proof of Theorem 2 can be met with equality unless $D\left(\;p_{Y}^{\;(\rho )}\;∥\;\;{\hat{p}}_{Y}^{\;(\rho )}\right)\to 0$, where $p_{Y}^{\;(\rho )}$ is the y-marginal PDF of $P_{X}p_{Y|X}^{\left(\rho \right)}$. This is similar to (2). Accordingly, since ${\hat{p}}_{Y}^{\;(\rho )}$ is Gaussian, while $p_{Y}^{\;(\rho )}$ is a convolution of $P_{X}$ with the Gaussian PDF $p_{Y|X}^{\left(\rho \right)}$, the type $P_{X}$ must converge to the Gaussian distribution with zero mean and variance $s^{2}$ in CF (it follows because the expression for the characteristic function of the zero-mean Gaussian distribution also has a Gaussian form) and CDF in order to achieve the exponents of Theorems 1 and 2. In both proofs the type $P_{X}$ represents the histograms of codewords, i.e., the empirical distributions of their quantized versions.*

The functions $E_{e}\left(R\right)$ and $E_{c}\left(R\right)$ given by (27) and (28) can be expressed parametrically [4,5,8] as follows:

**Proposition** **1** (Parametric representations of $E_{c}$ and $E_{e}$)**.** *For every $R\;\geq \;I(\;{\hat{p}}_{X},\;w)$ there exists a unique ρ∈(−1,0], such that*

(38) $$R\;=\;I\left(\;{\hat{p}}_{X},\;p_{Y|X}^{\left(\rho \right)}\right),\;\;\;\;\;\;E_{c}\left(R\right)\;=\;D\left(\;p_{Y|X}^{\left(\rho \right)}\;∥\;\;w\;\;|\;\;{\hat{p}}_{X}\right).$$

*For every $R\;\leq \;I(\;{\hat{p}}_{X},\;w)$ there exists a unique ρ≥0, such that*

(39) $$R\;=\;I\left(\;{\hat{p}}_{X},\;p_{Y|X}^{\left(\rho \right)}\right),\;\;\;\;\;\;E_{e}\left(R\right)\;=\;D\left(\;p_{Y|X}^{\left(\rho \right)}\;∥\;\;w\;\;|\;\;{\hat{p}}_{X}\right).$$

The correct-decoding exponent representation of (38) is equivalent to [5] [Equation (22)] and appears in [14] [Equations (25) and (26)], while the error exponent representation of (39) is equivalent to [4] [Equations (7.4.30) and (7.4.31)] and appears in [8] [Equations (70) and (71)] and [9] [Equations (329) and (330)]. Here we present an alternative proof of Proposition 1 in the vein of the proofs of Theorems 1 and 2.

**Proof.**  Let us denote $R_{\beta }≜I\left(\;{\hat{p}}_{X},\;p_{Y|X}^{\left(\beta \right)}\right)$. Then for β∈(−1,0] we can write a sandwich proof:

(40) $$\underset{\begin{array}{c} p_{Y|X}:\\ {\hat{p}}_{X}p_{Y|X}\;\in \;N \end{array}}{\inf}\left\{D\left(\;p_{Y|X}\;∥\;\;w\;\;|\;\;{\hat{p}}_{X}\right)\;+\;|\;R_{\beta }-I\left(\;{\hat{p}}_{X},\;p_{Y|X}\right)\;{|}^{+}\right\}$$

$$\begin{array}{cc} \overset{a}{\geq }\;\underset{-1\;<\;\rho \;\leq \;0}{\sup} & \underset{\begin{array}{c} p_{Y|X}:\\ {\hat{p}}_{X}p_{Y|X}\;\in \;N \end{array}}{\inf}\left\{D\left(\;p_{Y|X}\;∥\;\;w\;\;|\;\;{\hat{p}}_{X}\right)\;-\;\rho \left[R_{\beta }-D\left(\;p_{Y|X}\;∥\;\;{\hat{p}}_{Y}^{\;(\rho )}\;|\;\;{\hat{p}}_{X}\right)\right]\right\}\\ \overset{b}{\equiv }\;\underset{-1\;<\;\rho \;\leq \;0}{\sup} & \underset{\begin{array}{c} p_{Y|X}:\\ {\hat{p}}_{X}p_{Y|X}\;\in \;N \end{array}}{\inf}\left\{D\left(\;p_{Y|X}^{\left(\rho \right)}\;∥\;\;w\;\;|\;\;{\hat{p}}_{X}\right)\;-\;\rho \left[R_{\beta }-R_{\rho }\right]\;+\;\left(1+\rho \right)D\left(\;p_{Y|X}\;∥\;\;p_{Y|X}^{\left(\rho \right)}\;|\;\;{\hat{p}}_{X}\right)\right\} \end{array}$$

(41) $$\begin{array}{cc} \overset{c}{=}\;\underset{-1\;<\;\rho \;\leq \;0}{\sup}\; & \;\;\;\;\;\;\;\;\;\;\;\;\;\;\;\;\;\left\{D\left(\;p_{Y|X}^{\left(\rho \right)}\;∥\;\;w\;\;|\;\;{\hat{p}}_{X}\right)\;-\;\rho \left[R_{\beta }-R_{\rho }\right]\right\}\;\equiv \;E_{c}\left(R_{\beta }\right)\;\overset{d}{\geq }\;D\left(\;p_{Y|X}^{\left(\beta \right)}\;∥\;\;w\;\;|\;\;{\hat{p}}_{X}\right), \end{array}$$

where N denotes the set of all bivariate non-degenerate Gaussian PDF’s. Here (*a*) follows similarly to the inequality (*b*) in Theorem 2; (*b*) is an identity; (*c*) follows because ${\hat{p}}_{X}p_{Y|X}^{\left(\rho \right)}$ is Gaussian and $p_{Y|X}^{\left(\rho \right)}$ achieves the infimum; and (*d*) is a lower bound on the supremum at ρ=β. Finally, since the RHS of (41) is further lower-bounded by the infimum (40), we conclude that $E_{c}\left(R_{\beta }\right)=D\left(\;p_{Y|X}^{\left(\beta \right)}\;∥\;\;w\;\;|\;\;{\hat{p}}_{X}\right)$.

For β≥0, besides $R_{\beta }$ let us define $R_{\beta }^{\left(\rho \right)}≜D\left(\;p_{Y|X}^{\left(\rho \right)}\;∥\;\;{\hat{p}}_{Y}^{\;(\beta )}\;|\;\;{\hat{p}}_{X}\right)$. Then

(42) $$\underset{\begin{array}{c} p_{Y|X}:\\ {\hat{p}}_{X}p_{Y|X}\;\in \;N,\\ D(\;p_{Y|X}\;∥\;\;{\hat{p}}_{Y}^{\;(\beta )}\;\left|\;\;{\hat{p}}_{X}\right)\;\leq \;R_{\beta } \end{array}}{\inf}D\left(\;p_{Y|X}\;∥\;\;w\;\;|\;\;{\hat{p}}_{X}\right)$$

$$\begin{array}{ccc} \overset{a}{\geq } & \underset{\rho \;\geq \;0}{\sup}\underset{\begin{array}{c} p_{Y|X}:\\ {\hat{p}}_{X}p_{Y|X}\;\in \;N \end{array}}{\inf} & \left\{D\left(\;p_{Y|X}\;∥\;\;w\;\;|\;\;{\hat{p}}_{X}\right)\;+\;\rho \left[D\left(\;p_{Y|X}\;∥\;\;{\hat{p}}_{Y}^{\;(\beta )}\;|\;\;{\hat{p}}_{X}\right)-R_{\beta }\right]\right\}\\ \overset{b}{\equiv } & \underset{\rho \;\geq \;0}{\sup}\underset{\begin{array}{c} p_{Y|X}:\\ {\hat{p}}_{X}p_{Y|X}\;\in \;N \end{array}}{\inf} & \left\{D\left(\;p_{Y|X}^{\left(\rho \right)}\;∥\;\;w\;\;|\;\;{\hat{p}}_{X}\right)\;+\;\rho \left[R_{\beta }^{\left(\rho \right)}-R_{\beta }\right]\;+\;\left(1+\rho \right)D\left(\;p_{Y|X}\;∥\;\;p_{Y|X}^{\left(\rho \right)}\;|\;\;{\hat{p}}_{X}\right)\right\}\\ \overset{c}{=} & \underset{\rho \;\geq \;0}{\sup} & \left\{D\left(\;p_{Y|X}^{\left(\rho \right)}\;∥\;\;w\;\;|\;\;{\hat{p}}_{X}\right)\;+\;\rho \left[R_{\beta }^{\left(\rho \right)}-R_{\beta }\right]\right\} \end{array}$$

(43) $$\overset{d}{\geq }\underset{\rho \;\geq \;0}{\sup}\;\;\;\;\;\;\;\;\;\;\;\;\;\;\;\;\;\;\;\;\;\left\{D\left(\;p_{Y|X}^{\left(\rho \right)}\;∥\;\;w\;\;|\;\;{\hat{p}}_{X}\right)\;+\;\rho \left[R_{\rho }-R_{\beta }\right]\right\}\;\equiv \;E_{e}\left(R_{\beta }\right)\;\overset{e}{\geq }\;D\left(\;p_{Y|X}^{\left(\beta \right)}\;∥\;\;w\;\;|\;\;{\hat{p}}_{X}\right).$$

Here (*a*) follows due to the inequality under the first infimum; (*b*) is an identity; (*c*) follows because ${\hat{p}}_{X}p_{Y|X}^{\left(\rho \right)}$ is Gaussian and $p_{Y|X}^{\left(\rho \right)}$ achieves the infimum; and (*d*) follows because $R_{\beta }^{\left(\rho \right)}\geq R_{\rho }^{\left(\rho \right)}\equiv R_{\rho }$; (*e*) is a lower bound on the supremum at ρ=β. Since the RHS of (43) is lower-bounded by the infimum (42), we obtain $E_{e}\left(R_{\beta }\right)=D\left(\;p_{Y|X}^{\left(\beta \right)}\;∥\;\;w\;\;|\;\;{\hat{p}}_{X}\right)$. From $I\left(\;{\hat{p}}_{X},\;p_{Y|X}^{\left(\rho \right)}\right)=\frac{1}{2}\;\log_{b}\left(\sigma_{Y}^{2}\left(\rho \right)/\sigma_{Y|X}^{2}\left(\rho \right)\right)$ using (17) and (21) we obtain $\frac{dR_{\rho }}{d\rho }=\frac{dR_{\rho }}{dk_{\rho }}\cdot \frac{dk_{\rho }}{d\rho }<0$. Hence for every R>0 the parameter ρ(R)∈(−1,+∞) is unique. □

## 5. Method of Types

In this section we extend the method of types [1] to include the countable alphabets of uniformly spaced reals (9) by using power constraints on the types. The method of types in the form of the results of this section is then used in the rest of the paper. It allows us to establish converse bounds in terms of types in Section 6, Section 7 and Section 8 and is used in the Appendix B dedicated to the proof of Lemma 18 of Section 9, connecting between PDFs and types.

### 5.1. Alphabet Size

Consider all the types $P_{X}\in P_{n}\left(X_{n}\right)$ satisfying the power constraint $E_{P_{X}}\left[X^{2}\right]\leq c_{X}$. Let $X_{n}\left(c_{X}\right)\subseteq X_{n}$ denote the subset of the alphabet used by these types. In particular, every letter $x=i\Delta_{\alpha ,n}\in X_{n}\left(c_{X}\right)$ must satisfy $P_{X}\left(x\right)x^{2}\leq c_{X}$, while by the definition of a type we have $P_{X}\left(x\right)\geq 1/n$. This gives $|\;i\Delta_{\alpha ,n}\;|\;\leq \;\sqrt{nc_{X}}$. Then $X_{n}\left(c_{X}\right)$ is finite and by (7) we obtain

**Lemma** **2** (Alphabet size)**.** *$\;\;|\;X_{n}\left(c_{X}\right)\;|\;\;\leq \;\;2\sqrt{c_{X}}n^{1/2\;+\;\alpha }+1\;\;\leq \;\;\left(2\sqrt{c_{X}}+1\right)n^{1/2\;+\;\alpha }$.*

### 5.2. Size of a Type Class

For $P_{XY}\in P_{n}\left(X_{n}\times Y_{n}\right)$ let us define

$$\begin{array}{cc} S(P_{XY})\;\; & ≜\;\;\left\{\left(x,y\right)\in X_{n}\times Y_{n}:\;\;P_{XY}\left(x,y\right)>0\right\},\\ S(P_{X})\;\; & ≜\;\;\left\{x\in X_{n}:\;\;P_{X}\left(x\right)>0\right\},\;\;\;\;\;\;S\left(P_{Y}\right)\;\;≜\;\;\left\{y\in Y_{n}:\;\;P_{Y}\left(y\right)>0\right\}. \end{array}$$

**Lemma** **3** (Support of a joint type)**.** *Let $P_{XY}\in P_{n}\left(X_{n}\times Y_{n}\right)$ be a joint type, such that $E_{P_{X}}\left[X^{2}\right]\leq c_{X}$ and $E_{P_{Y}}\left[Y^{2}\right]\leq c_{Y}$. Then*

$$\begin{array}{cc} |\;S(P_{XY})\;|\;\; & \leq \;\;\sqrt{2\pi (c_{X}+c_{Y}+1/6)}\cdot n^{(1\;+\;\alpha \;+\;\beta )/2}. \end{array}$$

The proof is given in the Appendix A.

**Lemma** **4** (Support of a type)**.** *Let $P_{X}\in P_{n}\left(X_{n}\right)$ and $P_{Y}\in P_{n}\left(Y_{n}\right)$ be types, such that $E_{P_{X}}\left[X^{2}\right]\leq c_{X}$ and $E_{P_{Y}}\left[Y^{2}\right]\leq c_{Y}$. Then*

$$\begin{array}{cc} |\;S(P_{X})\;|\;\; & \leq \;\;{\left(12c_{X}+1\right)}^{1/3}\cdot n^{(1\;+\;2\alpha )/3},\\ |\;S(P_{Y})\;|\;\; & \leq \;\;{\left(12c_{Y}+1\right)}^{1/3}\cdot n^{(1\;+\;2\beta )/3}. \end{array}$$

The proof for $P_{X}$ is given in the Appendix A.

For $P_{Y}$, the parameters $c_{Y}$, β replace, respectively, $c_{X}$, α.

**Lemma** **5** (Size of a type class)**.** *Let $P_{XY}\in P_{n}\left(X_{n}\times Y_{n}\right)$ be a joint type, such that $E_{P_{X}}\left[X^{2}\right]\leq c_{X}$ and $E_{P_{Y}}\left[Y^{2}\right]\leq c_{Y}$. Then*

(44) $$\begin{array}{cc} H\left(P_{XY}\right)\;-\;c_{1}\;\frac{\log_{b}\;\left(n+1\right)}{n^{\gamma /2}}\;\; & \leq \;\;\frac{1}{n}\;\log_{b}\;\left|\;T\left(P_{XY}\right)\;\right|\;\;\leq \;\;H\left(P_{XY}\right), \end{array}$$

(45) $$\begin{array}{cc} H\left(P_{X}\right)\;-\;c_{2}\;\frac{\log_{b}\;\left(n+1\right)}{n^{2(1\;-\;\alpha )/3}}\;\; & \leq \;\;\frac{1}{n}\;\log_{b}\;\left|\;T\left(P_{X}\right)\;\right|\;\;\;\;\leq \;\;H\left(P_{X}\right), \end{array}$$

(46) $$\begin{array}{cc} H\left(P_{Y}\right)\;-\;c_{3}\;\frac{\log_{b}\;\left(n+1\right)}{n^{2(1\;-\;\beta )/3}}\;\; & \leq \;\;\frac{1}{n}\;\log_{b}\;\left|\;T\left(P_{Y}\right)\;\right|\;\;\;\;\;\leq \;\;H\left(P_{Y}\right), \end{array}$$

*where $c_{1}≜\sqrt{2\pi (c_{X}+c_{Y}+1/6)}$, $\;c_{2}≜{\left(12c_{X}+1\right)}^{1/3}$, and $c_{3}≜{\left(12c_{Y}+1\right)}^{1/3}$.*

**Proof.**  Observe that the standard type-size bounds (see, e.g., [6] [Lemma 2.3], Ref. [1] [Equation (11.16)]) can be rewritten as

(47) $$\frac{1}{{\left(n+1\right)}^{\left|\;S(P_{XY})\;\right|}}\;b^{\;nH(P_{XY})}\;\;\leq \;\;\left|\;T\left(P_{XY}\right)\;\right|\;\;\leq \;\;b^{\;nH(P_{XY})}.$$

Here $\left|\;S(P_{XY})\;\right|$ can be replaced with its upper bound of Lemma 3. This gives (44). The remaining bounds of (45) and (46) are obtained similarly using Lemma 4. □

Since it holds for any $y\in T(P_{Y})$ that $|\;T\left(P_{X|\;Y}\;|\;y\right)\;|\;=\;|\;T\left(P_{XY}\right)\;|\;/\;|\;T\left(P_{Y}\right)\;|$, and similarly for $x\in T(P_{X})$, as a corollary of the previous lemma we also obtain

**Lemma** **6** (Size of a conditional type class)**.** *Let $P_{XY}\in P_{n}\left(X_{n}\times Y_{n}\right)$ be a joint type, such that $E_{P_{X}}\left[X^{2}\right]\leq c_{X}$ and $E_{P_{Y}}\left[Y^{2}\right]\leq c_{Y}$. Then for $y\in T(P_{Y})$ and $x\in T(P_{X})$ respectively*

(48) $$\begin{array}{cc} H\left(X\;|\;Y\right)\;-\;c_{1}\;\frac{\log_{b}\;\left(n+1\right)}{n^{\gamma /2}}\;\; & \leq \;\;\frac{1}{n}\;\log_{b}\;\left|\;T\left(P_{X|\;Y}\;|\;y\right)\;\right|\;\;\leq \;\;H\left(X\;|\;Y\right)\;+\;c_{3}\;\frac{\log_{b}\;\left(n+1\right)}{n^{2(1\;-\;\beta )/3}}, \end{array}$$

(49) $$\begin{array}{cc} H\left(Y\;|\;X\right)\;-\;c_{1}\;\frac{\log_{b}\;\left(n+1\right)}{n^{\gamma /2}}\;\; & \leq \;\;\frac{1}{n}\;\log_{b}\;\left|\;T\left(P_{Y|X}\;|\;x\right)\;\right|\;\;\;\leq \;\;H\left(Y\;|\;X\right)\;+\;c_{2}\;\frac{\log_{b}\;\left(n+1\right)}{n^{2(1\;-\;\alpha )/3}}, \end{array}$$

*where $c_{1}$, $c_{2}$, and $c_{3}$ are defined as in Lemma 5.*

### 5.3. Number of Types

Let $P_{n}\left(X_{n},\;c_{X}\right)$ be the set of all the types $P_{X}\in P_{n}\left(X_{n}\right)$ satisfying the power constraint $E_{P_{X}}\left[X^{2}\right]\leq c_{X}$. Then its cardinality can be upper-bounded as follows:

(50) $$|\;P_{n}\left(X_{n},\;c_{X}\right)\;|\;\;\overset{\left(a\right)}{\leq }\;\;|\;P_{n}\left(X_{n}\left(c_{X}\right)\right)\;|\;\;\overset{\left(b\right)}{\leq }\;\;{\left(n+1\right)}^{|\;X_{n}\left(c_{X}\right)\;|}\;\;\overset{\left(c\right)}{\leq }\;\;{\left(n+1\right)}^{\left(2\sqrt{c_{X}}\;+\;1\right)n^{1/2\;+\;\alpha }},$$

where (*a*) follows by the definition of $X_{n}\left(c_{X}\right)$ preceding Lemma 2, (*b*) follows by [1] [Equation (11.6)], and (*c*) follows by Lemma 2. This bound is sub-exponential in *n* for α<1/2. This can be also further improved and made sub-exponential in *n* for all α∈(0,1) using Lemma 4, as follows.

**Lemma** **7** (Number of types)**.**

(51) $$|\;P_{n}\left(X_{n},\;c_{X}\right)\;|\;\;\leq \;\;{\left((n+1)c\right)}^{\tilde{c}\;n^{(1\;+\;2\alpha )/3}},$$

*where $c\;≜\;{\left(2\sqrt{c_{X}}+1\right)}^{1/(3/2\;+\;\alpha )}$ and $\tilde{c}\;≜\;\left(3/2+\alpha \right){\left(12c_{X}+1\right)}^{1/3}$.*

**Proof.**  Denoting $k\;≜\;|\;X_{n}\left(c_{X}\right)\;|$ and $\ell \;≜\;\max_{\;P_{X}\;\in \;P_{n}\left(X_{n},\;c_{X}\right)}\;\left|\;S\left(P_{X}\right)\;\right|$, we can upper-bound as follows

(52) |PnXn,cX|≤kℓ(n+1)ℓ≤kℓ(n+1)ℓ.

Substituting for *k* and *ℓ* their upper bounds of Lemma 2 (with n+1) and Lemma 4, we obtain (51). □

Similarly, let $P_{n}\left(X_{n}\times Y_{n},\;c_{X},\;c_{Y}\right)$ denote the set of all the joint types $P_{XY}\in P_{n}\left(X_{n}\times Y_{n}\right)$, such that $E_{P_{X}}\left[X^{2}\right]\leq c_{X}$ and $E_{P_{Y}}\left[Y^{2}\right]\leq c_{Y}$. Then its cardinality can be bounded as follows.

**Lemma** **8** (Number of joint types)**.**

(53) $$|\;P_{n}\left(X_{n}\times Y_{n},\;c_{X},\;c_{Y}\right)\;|\;\;\leq \;\;{\left((n+1)c\right)}^{\tilde{c}\;n^{(1\;+\;\alpha \;+\;\beta )/2}},$$

*where $\;c\;≜\;{\left[\left(2\sqrt{c_{X}}+1\right)\left(2\sqrt{c_{Y}}+1\right)\right]}^{1/(2\;+\;\alpha \;+\;\beta )}$ and $\;\tilde{c}\;≜\;\left(2+\alpha +\beta \right)\sqrt{2\pi (c_{X}+c_{Y}+1/6)}$.*

**Proof.**  Denoting $k\;≜\;|\;X_{n}\left(c_{X}\right)\;|\cdot |\;Y_{n}\left(c_{Y}\right)\;|$ and $\ell \;≜\;\max_{\;P_{XY}\;\in \;P_{n}\left(X_{n}\;\times \;Y_{n},\;c_{X},\;c_{Y}\right)}\;\left|\;S\left(P_{XY}\right)\;\right|$, we repeat the steps of (52) and use the bounds of Lemma 2 and Lemma 3 to obtain (53). □

## 6. Converse Lemma

In this section we prove a converse Lemma 10, which is then used both for the error exponent in Section 7 and for the correct-decoding exponent in Section 8.

In order to determine exponents in channel probabilities, it is convenient to take hold of the exponent in the channel probability *density*. Let $x=\left(x_{1},x_{2},\ldots ,x_{n}\right)\in R^{n}$ be a vector of *n* channel inputs and let $x^{q}=\left(x_{1}^{q},x_{2}^{q},\ldots ,x_{n}^{q}\right)\in X_{n}^{n}$ be its quantized version, with components

(54) $$x_{k}^{q}\;=\;Q_{\alpha }\left(x_{k}\right)\;≜\;\Delta_{\alpha ,n}\cdot \left\lfloor x_{k}/\Delta_{\alpha ,n}+1/2\right\rfloor ,\;\;\;\;\;\;k=1,\ldots ,n.$$

Similarly, let $y=\left(y_{1},y_{2},\ldots ,y_{n}\right)\in R^{n}$ be a vector of *n* channel outputs and let $y^{q}=\left(y_{1}^{q},y_{2}^{q},\ldots ,y_{n}^{q}\right)\in Y_{n}^{n}$ be its quantized version, with $y_{k}^{q}=Q_{\beta }\left(y_{k}\right)$ for all k=1,…,n. Then we have the following

**Lemma** **9** (PDF exponent)**.** *Let $x\in R^{n}$ and $y\in R^{n}$ be two channel input and output vectors, with their respective quantized versions $\left(x^{q},y^{q}\right)\in T\left(P_{XY}\right)$, such that $E_{P_{XY}}\left[{\left(Y-X\right)}^{2}\right]\leq c_{XY}$. Then*

$$\begin{array}{cc} & -\frac{1}{n}\;\log_{b}\;w\left(y^{q}\;|\;x^{q}\right)\;+\;\frac{\left(\Delta_{\alpha ,\;n}+\Delta_{\beta ,\;n}\right)\sqrt{c_{XY}}+{\left(\Delta_{\alpha ,\;n}+\Delta_{\beta ,\;n}\right)}^{2}/4}{2\sigma^{2}\ln b}\;\;\geq \;\;-\frac{1}{n}\;\log_{b}\;w\left(y\;|\;x\right)\\ \geq & -\frac{1}{n}\;\log_{b}\;w\left(y^{q}\;|\;x^{q}\right)\;-\;\frac{\left(\Delta_{\alpha ,\;n}+\Delta_{\beta ,\;n}\right)\sqrt{c_{XY}}}{2\sigma^{2}\ln b}. \end{array}$$

**Proof.**  The exponent can be equivalently rewritten as

(55) $$-\frac{1}{n}\;\log_{b}\;w\left(y\;|\;x\right)\;\;\equiv \;\;\log_{b}\;\left(\sigma \sqrt{2\pi }\right)\;+\;\frac{1}{2\sigma^{2}\ln b}\cdot \frac{1}{n}\sum_{k\;=\;1}^{n}{\left(y_{k}-x_{k}\right)}^{2}.$$

Defining $\delta_{k}\;≜\;\left(y_{k}-x_{k}\right)-\left(y_{k}^{q}-x_{k}^{q}\right)$, we observe that

(56) $$\frac{1}{n}\sum_{k\;=\;1}^{n}{\left(y_{k}-x_{k}\right)}^{2}\;\;=\;\;\frac{1}{n}\sum_{k\;=\;1}^{n}{\left(y_{k}^{q}-x_{k}^{q}\right)}^{2}\;+\;\frac{2}{n}\sum_{k\;=\;1}^{n}\left(y_{k}^{q}-x_{k}^{q}\right)\delta_{k}\;+\;\frac{1}{n}\sum_{k\;=\;1}^{n}\delta_{k}^{2}.$$

The second term on the RHS is bounded as:

(57) $$\begin{array}{cc} |\;\frac{2}{n}\sum_{k\;=\;1}^{n}\left(y_{k}^{q}-x_{k}^{q}\right)\delta_{k}\;|\;\; & \leq \;\;\frac{2}{n}\sum_{k\;=\;1}^{n}|\;y_{k}^{q}-x_{k}^{q}\;|\cdot |\;\delta_{k}\;|\;\;\overset{a}{\leq }\;\;\left(\Delta_{\alpha ,n}+\Delta_{\beta ,n}\right)\cdot \frac{1}{n}\sum_{k\;=\;1}^{n}\left|\;y_{k}^{q}-x_{k}^{q}\;\right|\\ \; & \overset{b}{\leq }\;\;\left(\Delta_{\alpha ,n}+\Delta_{\beta ,n}\right){\left[\frac{1}{n}\sum_{k\;=\;1}^{n}{\left(y_{k}^{q}-x_{k}^{q}\right)}^{2}\right]}^{1/2}\;\overset{c}{\leq }\;\;\left(\Delta_{\alpha ,n}+\Delta_{\beta ,n}\right)\sqrt{c_{XY}}, \end{array}$$

where (*a*) follows because $|\;\delta_{k}\;|\;\leq \;\left(\Delta_{\alpha ,n}+\Delta_{\beta ,n}\right)/2$, (*b*) follows by Jensen’s inequality for the concave (∩) function $f\left(t\right)=\sqrt{t}$, and (*c*) follows by the condition of the lemma. The third term is bounded as

(58) $$\frac{1}{n}\sum_{k\;=\;1}^{n}\delta_{k}^{2}\;\;\leq \;\;{\left(\Delta_{\alpha ,n}+\Delta_{\beta ,n}\right)}^{2}/4.$$

Since the exponent with the quantized versions $-\frac{1}{n}\;\log_{b}\;w\left(y^{q}\;|\;x^{q}\right)$, in turn, can also be rewritten similarly to (55), the result of the lemma follows by (55)–(58). □

The following lemma will be used both for the upper bound on the error exponent and for the lower bound on the correct-decoding exponent.

**Lemma** **10** (Conditional probability of correct decoding)**.** *Let $P_{XY}\in P_{n}\left(X_{n}\times Y_{n}\right)$ be a joint type, such that $E_{P_{X}}\left[X^{2}\right]\leq c_{X}$, $E_{P_{Y}}\left[Y^{2}\right]\leq c_{Y}$, and $E_{P_{XY}}\left[{\left(Y-X\right)}^{2}\right]\leq c_{XY}$, and let C be a codebook, such that the quantized versions (54) of its codewords x(m), m=1,2,…,M(n,R), are all of the type $P_{X}$, that is:*

$$x^{q}\left(m\right)\;=\;Q_{\alpha }\left(x\left(m\right)\right)\;=\;\left(Q_{\alpha }\left(x_{1}\left(m\right)\right),Q_{\alpha }\left(x_{2}\left(m\right)\right),\ldots ,\;Q_{\alpha }\left(x_{n}\left(m\right)\right)\right)\;\in \;T\left(P_{X}\right),\;\;\;\forall m.$$

*Let $J∼Unif\;\left(\{1,2,\ldots ,\;M\}\right)$ be a random variable, independent of the channel noise, and let x(J)→Y be the random channel-input and channel-output vectors, respectively. Let $Y^{q}=Q_{\beta }\left(Y\right)\in Y_{n}^{n}$. Then*

$$\mathrm{Pr}\left\{g\left(Y\right)=J\;|\;\left(x^{q}\left(J\right),\;Y^{q}\right)\;\in \;T\left(P_{XY}\right)\right\}\;\;\leq \;\;b^{\;-n\left(\tilde{R}\;-\;I\left(P_{XY}\right)\;+\;o\left(1\right)\right)},$$

*where $\tilde{R}=\frac{1}{n}\;\log_{b}\;M\left(n,R\right)$, and o(1)→0, as n→∞, depending only on α, β, $c_{X}$, $c_{Y}$, $c_{XY}$, and $\sigma^{2}$.*

**Proof.**  First, from the single code (C,g) we create an ensemble of codes, where each member code has the same probability of error/correct-decoding as the original code (C,g). Then we upper bound the ensemble average probability of correct decoding.

Considering the codebook C as an M×n matrix, we permute its *n* columns. This produces a set of codebooks: $C_{\ell }$, ℓ=1,…,n!. The quantized versions of all the codewords of each codebook $C_{\ell }$ belong to the same type class $T(P_{X})$. In accordance with $C_{\ell }$, we permute also the *n* coordinates of each $y\in D_{m}$ of the decision regions $D_{m}$ in the definition (6) of the decoder *g*, obtaining open sets $D_{m}^{\left(\ell \right)}$ and creating in this way an ensemble of codes $\left(C_{\ell },g_{\ell }\right)$, ℓ=1,…,n!.

Let $x_{\ell }\left(J\right)\to Y_{\ell }$ denote the random channel-input and channel-output vectors, respectively, when using the code with an index ℓ∈{1,…,n!}. Let $x_{\ell }^{q}\left(J\right)$ and $Y_{\ell }^{q}$ denote their respective quantized versions. Since the additive channel noise is i.i.d., permutation of components does not change the distribution of the noise vector $Y_{\ell }-x_{\ell }\left(J\right)$, and we obtain

(59) $$\begin{array}{cc} \mathrm{Pr}\left\{g\left(Y\right)=J,\;\left(x^{q}\left(J\right),\;Y^{q}\right)\;\in \;T\left(P_{XY}\right)\right\}\; & =\;\mathrm{Pr}\left\{g_{\ell }\left(Y_{\ell }\right)=J,\;\left(x_{\ell }^{q}\left(J\right),\;Y_{\ell }^{q}\right)\;\in \;T\left(P_{XY}\right)\right\},\;\;\;\forall \ell , \end{array}$$

(60) $$\begin{array}{cc} \mathrm{Pr}\left\{\left(x^{q}\left(J\right),\;Y^{q}\right)\;\in \;T\left(P_{XY}\right)\right\}\; & =\;\mathrm{Pr}\left\{\left(x_{\ell }^{q}\left(J\right),\;Y_{\ell }^{q}\right)\;\in \;T\left(P_{XY}\right)\right\},\;\;\;\forall \ell . \end{array}$$

Suppose that one of the codes $\left(C_{\ell },g_{\ell }\right)$, ℓ=1,…,n!, is used for communication with probability 1/n!, chosen independently of the sent message *J* and of the channel noise. Let $L∼\mathrm{Unif}\;\left(\{1,2,\ldots ,\;n!\}\right)$ be the random variable denoting the index of this code. Then, using (59) and (60) we obtain

(61) $$\begin{array}{cc} \mathrm{Pr}\left\{g\left(Y\right)=J\;|\;\left(x^{q}\left(J\right),\;Y^{q}\right)\;\in \;T\left(P_{XY}\right)\right\}\; & =\;\mathrm{Pr}\left\{g_{L}\left(Y_{L}\right)=J\;|\;\left(x_{L}^{q}\left(J\right),\;Y_{L}^{q}\right)\;\in \;T\left(P_{XY}\right)\right\}. \end{array}$$

In what follows, we upper bound the RHS of (61) with an added condition that $Y_{L}^{q}=y\in T\left(P_{Y}\right)$:

$$\mathrm{Pr}\left\{\;g_{L}\left(Y_{L}\right)=J\;\;|\;\;x_{L}^{q}\left(J\right)\in T\left(P_{X|\;Y}\;|\;y\right),\;Y_{L}^{q}=y\right\}.$$

The total number of codes in the ensemble can be rewritten as

(62) $$n!\;\;=\;\;|\;T\left(P_{X}\right)\;|\prod_{x\;\in \;S(P_{X})}\left(nP_{X}\left(x\right)\right)!\;\;≜\;\;\left|\;T\left(P_{X}\right)\;\right|\cdot \Pi \left(P_{X}\right).$$

Given $y\in T(P_{Y})$, the total number of all the codewords in the ensemble such that their quantized versions belong to the same conditional type class $T(P_{X|\;Y}\;|\;y)$ (counted as distinct if the codewords belong to different ensemble member codes or represent different messages) is given by

(63) $$S\;=\;\underset{\mathrm{for}\;a\;\mathrm{message}\;m}{\underset{︸}{|\;T\left(P_{X|\;Y}\;|\;y\right)\;|\;\cdot \;\Pi \left(P_{X}\right)}}\;\;\cdot \;\;M.$$

Let N(ℓ) denote the number of the codewords in a codebook $C_{\ell }$ such that their quantized versions belong to $T(P_{X|\;Y}\;|\;y)$. Given that $Y_{L}^{q}=y$, the channel output vector $Y_{L}$ falls into a hypercube region of $R^{n}$:

$$B\;\;≜\;\;\{\tilde{y}\in R^{n}:\;Q_{\beta }\left(\tilde{y}\right)=y\}.$$

For any $x\in R^{n}$ such that $Q_{\alpha }\left(x\right)\in T\left(P_{X|\;Y}\;|\;y\right)$ and any open region $D\subseteq R^{n}$, by Lemma 9 we obtain

$$\begin{array}{cc} \; & b^{n\left(E_{P_{XY}}\left[\;\log_{b}\;w\left(Y\;|\;X\right)\;\right]\;+\;o_{1}\left(1\right)\right)}\cdot \mathrm{vol}\left(B\cap D\right)\;\;\geq \;\;\mathrm{Pr}\left\{Y_{L}\in B\cap D\;|\;x_{L}\left(J\right)=x\right\} \end{array}$$

(64) $$\begin{array}{cc} \geq & b^{n\left(E_{P_{XY}}\left[\;\log_{b}\;w\left(Y\;|\;X\right)\;\right]\;+\;o_{2}\left(1\right)\right)}\cdot \mathrm{vol}\left(B\cap D\right). \end{array}$$

Then, since all the codes and messages are equiprobable, the conditional probability of the code with the index *ℓ* is upper-bounded as

(65) $$\mathrm{Pr}\left\{\;L=\ell \;\;|\;\;x_{L}^{q}\left(J\right)\in T\left(P_{X|\;Y}\;|\;y\right),\;Y_{L}^{q}=y\right\}\;\;\leq \;\;b^{\;n\left(o_{1}\left(1\right)\;-\;o_{2}\left(1\right)\right)}N\left(\ell \right)/S.$$

For N(ℓ)>0, let $m_{1}<m_{2}<\ldots <m_{N(\ell )}$ be the indices of all the codewords in the codebook $C_{\ell }$ with their quantized versions in $T(P_{X|\;Y}\;|\;y)$. Given that indeed the codebook $C_{\ell }$ has been used for communication, similarly to (65), by (64) the conditional probability of correct decoding can be upper-bounded as

(66) $$\begin{array}{cc} \mathrm{Pr}\left\{\;g_{\ell }\left(Y_{\ell }\right)=J\;|\;x_{\ell }^{q}\left(J\right)\in T\left(P_{X|\;Y}\;|\;y\right),\;Y_{\ell }^{q}=y,\;L=\ell \right\}\;\; & \leq \;\sum_{j\;=\;1}^{N(\ell )}\frac{\mathrm{vol}\left(B\cap D_{m_{j}}^{\left(\ell \right)}\right)b^{\;n\;\cdot \;o(1)}}{N(\ell )\mathrm{vol}(B)}\;\leq \;\frac{b^{\;n\;\cdot \;o(1)}}{N(\ell )}, \end{array}$$

where the second inequality follows because the decision regions $D_{m_{j}}^{\left(\ell \right)}$ are disjoint. Summing up over all the codes, we finally obtain:

$$\begin{array}{cc} \; & \mathrm{Pr}\left\{\;g_{L}\left(Y_{L}\right)=J\;\;|\;\;x_{L}^{q}\left(J\right)\in T\left(P_{X|\;Y}\;|\;y\right),\;Y_{L}^{q}=y\right\}\;\;\overset{a}{\leq }\;\;\sum_{\begin{array}{c} 1\;\leq \;\ell \;\leq \;n!\;:\;N(\ell )\;>\;0 \end{array}}\frac{N(\ell )}{S}\cdot \frac{1}{N(\ell )}\cdot b^{\;n\;\cdot \;o(1)}\\ =\;\; & \sum_{\begin{array}{c} 1\;\leq \;\ell \;\leq \;n!\;:\;N(\ell )\;>\;0 \end{array}}\frac{1}{S}\cdot b^{\;n\;\cdot \;o(1)}\;\;\leq \;\;\frac{n!}{S}\cdot b^{\;n\;\cdot \;o(1)}\;\;\overset{b}{=}\;\;\frac{\left|\;T(P_{X})\;\right|}{|\;T(P_{X|\;Y}\;|\;y)\;|\;\;M}\cdot b^{\;n\;\cdot \;o(1)}\;\;\overset{c}{\leq }\;\;b^{\;-n\left(\tilde{R}\;-\;I\left(P_{XY}\right)\;+\;o\left(1\right)\right)}, \end{array}$$

where (*a*) follows by (65) and (66), (*b*) follows by (62) and (63), and (*c*) follows by (45) of Lemma 5 and (48) of Lemma 6. □

In the next two sections, we derive converse bounds on the error and correct-decoding exponents in terms of types.

## 7. Error Exponent

The end result of this section is given by Lemma 13 and represents a converse bound on the error exponent by the method of types.

**Lemma** **11** (Error exponent of mono-composition codebooks)**.** *Let $P_{X}\in P_{n}\left(X_{n}\right)$ be a type, such that $E_{P_{X}}\left[X^{2}\right]\leq c_{X}$, and let C be a codebook, such that the quantized versions (54) of its codewords x(m), m=1,2,…,M(n,R), are all of the type $P_{X}$, that is:*

$$x^{q}\left(m\right)\;=\;Q_{\alpha }\left(x\left(m\right)\right)\;=\;\left(Q_{\alpha }\left(x_{1}\left(m\right)\right),Q_{\alpha }\left(x_{2}\left(m\right)\right),\ldots ,\;Q_{\alpha }\left(x_{n}\left(m\right)\right)\right)\;\in \;T\left(P_{X}\right),\;\;\;\forall m.$$

*Let $J∼Unif\;\left(\{1,2,\ldots ,\;M\}\right)$ be a random variable, independent of the channel noise, and let x(J)→Y be the random channel-input and channel-output vectors, respectively. Then for any parameter $c_{XY}$*

(67) $$-\frac{1}{n}\log_{b}\mathrm{Pr}\left\{g(Y)\neq J\right\}\;\;\leq \;\;\min_{\begin{array}{c} P_{Y|X}:\\ P_{XY}\;\in \;P_{n}\left(X_{n}\;\times \;Y_{n}\right),\\ E\left[{\left(Y-X\right)}^{2}\right]\;\leq \;c_{XY},\\ I\left(P_{X},\;P_{Y|X}\right)\;\leq \;\tilde{R}\;-\;o\left(1\right) \end{array}}\left\{D\left(P_{Y|X}\;∥\;W_{n}\;|\;P_{X}\right)\right\}\;+\;o\left(1\right),$$

*where $\tilde{R}=\frac{1}{n}\;\log_{b}\;M\left(n,R\right)$, and o(1)→0, as n→∞, depending only on α, β, $c_{X}$, $c_{XY}$, and $\sigma^{2}$.*

**Proof.**  For a joint type $P_{XY}\in P_{n}\left(X_{n}\times Y_{n}\right)$ with the marginal type $P_{X}$, such that $E_{P_{XY}}\left[{\left(Y-X\right)}^{2}\right]\leq c_{XY}$, we have also

$$E_{P_{Y}}\left[Y^{2}\right]\;\;\leq \;\;{\left(E_{P_{X}}^{1/2}\left[X^{2}\right]\;+\;E_{P_{XY}}^{1/2}\left[{\left(Y-X\right)}^{2}\right]\right)}^{2}\;\;\leq \;\;{\left(\sqrt{c_{X}}\;+\;\sqrt{c_{XY}}\right)}^{2}\;\;≜\;\;c_{Y}.$$

Then with $\;Y^{q}=Q_{\beta }\left(Y\right)\in Y_{n}^{n}\;$ for any 1≤j≤M, we obtain

(68) $$\begin{array}{cc} \mathrm{Pr}\left\{\left(x^{q}\left(J\right),\;Y^{q}\right)\;\in \;T\left(P_{XY}\right)\;|\;J=j\right\}\;\; & \overset{a}{\geq }\;\;|\;T\left(P_{Y|X}\;|\;x^{q}\left(j\right)\right)\;|\cdot \Delta_{\beta ,\;n}^{n}\cdot b^{\;n\left(E_{P_{XY}}\left[\;\log_{b}\;w\left(Y\;|\;X\right)\;\right]\;+\;o\left(1\right)\right)}\\ \; & \overset{b}{\geq }\;\;b^{\;-n\left(D(P_{Y|X}\;∥\;W_{n}\;|\;P_{X})\;+\;o\left(1\right)\right)},\;\;\;\;\;\;\forall j, \end{array}$$

where (*a*) follows by Lemma 9, and (*b*) follows by (49) of Lemma 6 and (10). This gives

(69) $$\begin{array}{cc} \mathrm{Pr}\left\{\left(x^{q}\left(J\right),\;Y^{q}\right)\;\in \;T\left(P_{XY}\right)\right\}\;\; & \geq \;\;b^{\;-n\left(D(P_{Y|X}\;∥\;W_{n}\;|\;P_{X})\;+\;o\left(1\right)\right)}. \end{array}$$

Now we are ready to apply Lemma 10:

$$\begin{array}{cc} \mathrm{Pr}\left\{g(Y)\neq J\right\}\;\; & \geq \;\;\mathrm{Pr}\left\{\left(x^{q}\left(J\right),\;Y^{q}\right)\;\in \;T\left(P_{XY}\right)\right\}\cdot \mathrm{Pr}\left\{g\left(Y\right)\neq J\;|\;\left(x^{q}\left(J\right),\;Y^{q}\right)\;\in \;T\left(P_{XY}\right)\right\}\\ \; & \overset{a}{\geq }\;\;b^{\;-n\left(D(P_{Y|X}\;∥\;W_{n}\;|\;P_{X})\;+\;o\left(1\right)\right)}\cdot \left[1\;-\;b^{\;-n\left(\tilde{R}\;-\;I\left(P_{X},\;P_{Y|X}\right)\;+\;o\left(1\right)\right)}\right]\\ \; & \overset{b}{\geq }\;\;b^{\;-n\left(D(P_{Y|X}\;∥\;W_{n}\;|\;P_{X})\;+\;o\left(1\right)\right)}\cdot 1/2, \end{array}$$

where (*a*) follows by (69) and Lemma 10, and (*b*) holds for $I\left(P_{X},P_{Y|X}\right)\;\leq \;\tilde{R}-\log_{b}\left(2\right)/n+o\left(1\right)$. □

**Lemma** **12** (Type constraint)**.** *For any ϵ>0 there exists $n_{0}=n_{0}\left(\alpha ,s^{2},\epsilon \right)\in N$, such that for any $n>n_{0}$ and any codeword $x\in R^{n}$, satisfying the power constraint (5), the quantized version of that codeword, defined by (54), satisfies the power constraint (5) within ϵ, that is with $s^{2}$ replaced by $s^{2}+\epsilon$.*

The proof is the same as (56)–(58).

**Lemma** **13** (Error exponent)**.** *Let $J∼Unif\;\left(\{1,2,\ldots ,\;M\}\right)$ be a random variable, independent of the channel noise, and let x(J)→Y be the random channel-input and channel-output vectors, respectively. Then for any $c_{XY}$ and ϵ>0 there exists $n_{0}=n_{0}\left(\alpha ,\;\beta ,\;s^{2},\;\sigma^{2},\;c_{XY},\;\epsilon \right)\in N$, such that for any $n>n_{0}$*

(70) $$-\frac{1}{n}\;\log_{b}\mathrm{Pr}\left\{g(Y)\neq J\right\}\;\;\leq \;\;\max_{\begin{array}{c} P_{X|}:\\ P_{X}\;\in \;P_{n}\left(X_{n}\right),\\ E\left[X^{2}\right]\;\leq \;s^{2}\;+\;\epsilon \end{array}}\;\;\min_{\begin{array}{c} P_{Y|X}:\\ P_{XY}\;\in \;P_{n}\left(X_{n}\;\times \;Y_{n}\right),\\ E\left[{\left(Y-X\right)}^{2}\right]\;\leq \;c_{XY},\\ I(P_{X},\;P_{Y|X})\;\leq \;R\;-\;\epsilon \end{array}}\left\{D\left(P_{Y|X}\;∥\;W_{n}\;|\;P_{X}\right)\right\}\;+\;o\left(1\right),$$

*where o(1)→0, as n→∞, depending only on the parameters α, β, $s^{2}+\epsilon$, $c_{XY}$, and $\sigma^{2}$.*

**Proof.**  For a type $P_{X}\in P_{n}\left(X_{n}\right)$ let us define $\;M\left(P_{X}\right)\;≜\;|\;\left\{j:\;x^{q}\left(j\right)\in T\left(P_{X}\right)\right\}\;|$. Then for any *n* greater than $n_{0}$ of Lemma 12, there exists at least one type $P_{X}$ such that

(71) $$M\left(P_{X}\right)\;\;\geq \;\;\frac{M}{\left|\;P_{n}\left(X_{n},\;s^{2}+\epsilon \right)\;\right|}\;\;\geq \;\;\frac{M}{{\left((n+1)c\right)}^{\tilde{c}\;n^{(1\;+\;2\alpha )/3}}},$$

where the second inequality follows by Lemma 7 applied with $c_{X}=s^{2}+\epsilon$. Then we can use such a type for a bound:

$$\begin{array}{c} \mathrm{Pr}\left\{g(Y)\neq J\right\}\;\;=\;\;\frac{1}{M}\sum_{j\;=\;1}^{M}\mathrm{Pr}\left\{Y\notin D_{J}\;|\;J=j\right\}\;\;\geq \;\;\frac{1}{M}\sum_{\begin{array}{c} 1\;\leq \;j\;\leq \;M\;:\\ x^{q}\left(j\right)\;\in \;T\left(P_{X}\right) \end{array}}\mathrm{Pr}\left\{Y\notin D_{J}\;|\;J=j\right\}\\ \overset{a}{\geq }\;b^{\;n\;\cdot \;o(1)}\;\frac{1}{M(P_{X})}\sum_{\begin{array}{c} 1\;\leq \;j\;\leq \;M\;:\\ x^{q}\left(j\right)\;\in \;T\left(P_{X}\right) \end{array}}\mathrm{Pr}\left\{Y\notin D_{J}\;|\;J=j\right\} \end{array}$$

(72) $$\;\;\;\;\;\;\;\;\;\;\;\;\;\;\;\;\;\;\;\;\;\;\;\;\;\;\overset{b}{=}\;\;b^{\;n\;\cdot \;o(1)}\;\mathrm{Pr}\left\{\;\tilde{g}\left(\tilde{Y}\right)\;\neq \;\tilde{J}\;\right\},$$

where (*a*) follows by (71), and (*b*) holds for the random variable $\tilde{J}∼\mathrm{Unif}\;\left(\{1,2,\ldots ,\;M\left(P_{X}\right)\}\right)$, independent of the channel noise, and the channel input/output random vectors $x\left(m_{\tilde{J}}\right)\to \tilde{Y}$ with the decoder

$$\tilde{g}\left(y\right)\;\;≜\;\;\left\{\begin{array}{cc} 0, & \;\;\;y\in \overset{\;M(P_{X})}{\underset{\;j\;=\;1}{⋂}}D_{m_{j}}^{c},\\ j, & \;\;\;y\in D_{m_{j}},\;\;\;j\in \left\{1,2,\ldots ,\;M\left(P_{X}\right)\right\}, \end{array}\right.$$

where $m_{1}<\ldots <m_{M(P_{X})}$ are the indices of the codewords in C with their quantized versions in $T(P_{X})$.

It follows now from (72) that the LHS of (70) can be upper-bounded by (67) of Lemma 11 with $\tilde{R}=\frac{1}{n}\;\log_{b}\;M\left(P_{X}\right)$. Substituting then (71) in place of $M(P_{X})$ we obtain a stricter condition under the minimum of (67), leading to an upper bound with a condition $I\left(P_{X},P_{Y|X}\right)\;\leq \;R-o\left(1\right)$ and to (70). □

## 8. Correct-Decoding Exponent

The end result of this section is Lemma 17, which is a converse bound on the correct-decoding exponent by the method of types.

**Lemma** **14** (Joint type constraint)**.** *For any ϵ>0 and ${\tilde{\sigma }}^{2}$, there exists $n_{0}=n_{0}\left(\alpha ,\;\beta ,\;{\tilde{\sigma }}^{2},\;\epsilon \right)\in N$, such that for any $n>n_{0}$ and any pair of vectors $x,y\in R^{n}$ satisfying*

$$\frac{1}{n}\sum_{k\;=\;1}^{n}{\left(y_{k}-x_{k}\right)}^{2}\;\;\leq \;\;{\tilde{\sigma }}^{2},$$

*the respective quantized versions $x^{q}=Q_{\alpha }\left(x\right)$ and $y^{q}=Q_{\beta }\left(y\right)$, defined as in (54), satisfy*

$$\frac{1}{n}\sum_{k\;=\;1}^{n}{\left(y_{k}^{q}-x_{k}^{q}\right)}^{2}\;\;\leq \;\;{\tilde{\sigma }}^{2}\;+\;\epsilon .$$

The proof is the same as (56)–(58).

We use a Chernoff bound for the probability of an event when the method of types cannot be applied:

**Lemma** **15** (Chernoff bound)**.** *Let $Z_{k}∼N\left(0,\sigma^{2}\right)$, k=1,2,…,n, be n independent random variables. Then for $\;{\tilde{\sigma }}^{2}\geq \sigma^{2}\;$ and $\;f\left(x\right)\;≜\;\frac{1}{2}\left[x-1-\ln(x)\right]$:*

$$\mathrm{Pr}\left\{\frac{1}{n}\sum_{k\;=\;1}^{n}Z_{k}^{2}\;\geq \;{\tilde{\sigma }}^{2}\right\}\;\;\leq \;\;\exp\left\{-nf({\tilde{\sigma }}^{2}/\sigma^{2})\right\}.$$

**Lemma** **16** (Correct-decoding exponent of mono-composition codebooks)**.** *Let $P_{X}\in P_{n}\left(X_{n}\right)$ be a type, such that $E_{P_{X}}\left[X^{2}\right]\leq c_{X}$, and let C be a codebook, such that the quantized versions (54) of its codewords x(m), m=1,2,…,M(n,R), are all of the type $P_{X}$, that is:*

$$x^{q}\left(m\right)\;=\;Q_{\alpha }\left(x\left(m\right)\right)\;=\;\left(Q_{\alpha }\left(x_{1}\left(m\right)\right),Q_{\alpha }\left(x_{2}\left(m\right)\right),\ldots ,\;Q_{\alpha }\left(x_{n}\left(m\right)\right)\right)\;\in \;T\left(P_{X}\right),\;\;\;\forall m.$$

*Let $J∼Unif\;\left(\{1,2,\ldots ,\;M\}\right)$ be a random variable, independent of the channel noise, and let x(J)→Y be the random channel-input and channel-output vectors, respectively. Then for any ϵ>0 and $\;{\tilde{\sigma }}^{2}\geq \sigma^{2}$ there exists $n_{0}=n_{0}\left(\alpha ,\;\beta ,\;{\tilde{\sigma }}^{2},\;\epsilon \right)\in N$, such that for any $n>n_{0}$*

(73) $$-\frac{1}{n}\log_{b}\mathrm{Pr}\left\{g(Y)=J\right\}\geq \min\left\{E_{n}\left(P_{X},\;R,\;\tilde{\sigma },\;\epsilon \right),\;E\left(\tilde{\sigma }\right)\right\}\;+\;o\left(1\right),$$

(74) $$E(\tilde{\sigma })\;\;≜\;\;\frac{1}{2\ln b}\left[\;{\tilde{\sigma }}^{2}/\sigma^{2}\;-\;1\;-\;\ln\;\left({\tilde{\sigma }}^{2}/\sigma^{2}\right)\;\right],$$

(75) $$E_{n}\left(P_{X},\;R,\;\tilde{\sigma },\;\epsilon \right)\;\;≜\;\;\min_{\begin{array}{c} P_{Y|X}:\\ P_{XY}\;\in \;P_{n}\left(X_{n}\;\times \;Y_{n}\right),\\ E\left[{\left(Y-X\right)}^{2}\right]\;\leq \;{\tilde{\sigma }}^{2}\;+\;\epsilon \end{array}}\left\{D\left(P_{Y|X}\;∥\;W_{n}\;|\;P_{X}\right)\;+\;|\;R\;-\;I\left(P_{X},P_{Y|X}\right)\;{|}^{+}\right\},$$

*where ${\left|\;t\;\right|}^{+}≜\max\;\left\{0,t\right\}$, and o(1)→0, as n→∞, depending only on α, β, $c_{X}$, ${\tilde{\sigma }}^{2}+\epsilon$, and $\sigma^{2}$.*

**Proof.**  We consider probabilities of two disjoint events:

(76) $$\begin{array}{lll} \mathrm{Pr}\left\{g(Y)=J\right\} & \leq & \mathrm{Pr}\left\{g\left(Y\right)={J,\;∥Y-x\left(J\right)∥}^{2}\leq n{\tilde{\sigma }}^{2}\right\}\;+\;\mathrm{Pr}\left\{{∥Y-x\left(J\right)∥}^{2}>n{\tilde{\sigma }}^{2}\right\}\\ & \leq & 2\max\left\{\mathrm{Pr}\left\{g\left(Y\right)={J,\;∥Y-x\left(J\right)∥}^{2}\leq n{\tilde{\sigma }}^{2}\right\},\;\;\mathrm{Pr}\left\{{∥Y-x\left(J\right)∥}^{2}\geq n{\tilde{\sigma }}^{2}\right\}\right\}. \end{array}$$

For the first term in the maximum, we obtain:

$$\begin{array}{c} \mathrm{Pr}\left\{g\left(Y\right)={J,\;∥Y-x\left(J\right)∥}^{2}\leq n{\tilde{\sigma }}^{2}\right\}\;\;\overset{a}{\leq }\;\;\mathrm{Pr}\left\{g\left(Y\right)=J,\;∥Y^{q}-x^{q}{\left(J\right)∥}^{2}\leq n\left({\tilde{\sigma }}^{2}+\epsilon \right)\right\}\\ \;\;\;\;\;\;\;\;\;\;\;\;\;\;\;\;\;\;\;\;\;\;\;\;\;\;\;\;\;\;\;\;\;\;\;\overset{b}{=}\sum_{\begin{array}{c} P_{Y|X}:\\ P_{XY}\;\in \;P_{n}\left(X_{n}\;\times \;Y_{n}\right),\\ E\left[{\left(Y-X\right)}^{2}\right]\;\leq \;{\tilde{\sigma }}^{2}\;+\;\epsilon \end{array}}\mathrm{Pr}\left\{g\left(Y\right)=J,\;\left(x^{q}\left(J\right),\;Y^{q}\right)\;\in \;T\left(P_{XY}\right)\right\} \end{array}$$

(77) $$\overset{c}{\leq }\;\;|\;P_{n}\left(X_{n}\times Y_{n},\;c_{X},\;c_{Y}\right)\;|\max_{\begin{array}{c} P_{Y|X}:\\ P_{XY}\;\in \;P_{n}\left(X_{n}\;\times \;Y_{n}\right),\\ E\left[{\left(Y-X\right)}^{2}\right]\;\leq \;{\tilde{\sigma }}^{2}\;+\;\epsilon \end{array}}\mathrm{Pr}\left\{g\left(Y\right)=J,\;\left(x^{q}\left(J\right),\;Y^{q}\right)\;\in \;T\left(P_{XY}\right)\right\},$$

where (*a*) holds with $Y^{q}=Q_{\beta }\left(Y\right)\in Y_{n}^{n}\;$ for all $n>n_{0}\left(\alpha ,\;\beta ,\;{\tilde{\sigma }}^{2},\;\epsilon \right)$ of Lemma 14, (*b*) holds because $x^{q}\left(J\right)\in T\left(P_{X}\right)$, and in (*c*) we use the notation $P_{n}\left(X_{n}\times Y_{n},\;c_{X},\;c_{Y}\right)$ for the set of all the joint types $P_{XY}\in P_{n}\left(X_{n}\times Y_{n}\right)$ satisfying both $E_{P_{X}}\left[X^{2}\right]\leq c_{X}$ and $E_{P_{Y}}\left[Y^{2}\right]\leq {\left(\sqrt{c_{X}}+\sqrt{{\tilde{\sigma }}^{2}+\epsilon }\right)}^{2}≜c_{Y}$. By the same steps as in (68), we further obtain

(78) $$\begin{array}{cc} \mathrm{Pr}\left\{\left(x^{q}\left(J\right),\;Y^{q}\right)\;\in \;T\left(P_{XY}\right)\right\}\;\; & \leq \;\;b^{\;-n\left(D(P_{Y|X}\;∥\;W_{n}\;|\;P_{X})\;+\;o\left(1\right)\right)}, \end{array}$$

while Lemma 10 gives

(79) $$\mathrm{Pr}\left\{g\left(Y\right)=J\;|\;\left(x^{q}\left(J\right),\;Y^{q}\right)\;\in \;T\left(P_{XY}\right)\right\}\;\;\leq \;\;b^{\;-n\left(|\;R\;-\;I\left(P_{XY}\right){\;|}^{+}\;+\;o\left(1\right)\right)}.$$

Now by Lemma 8 for the number of joint types and by (77)–(79), we obtain

$$\mathrm{Pr}\left\{g\left(Y\right)={J,\;∥Y-x\left(J\right)∥}^{2}\leq n{\tilde{\sigma }}^{2}\right\}\;\;\leq \;\;b^{\;-n\left(E_{n}\left(P_{X},\;R,\;\tilde{\sigma },\;\epsilon \right)\;+\;o\left(1\right)\right)},$$

where $E_{n}\left(P_{X},\;R,\;\tilde{\sigma },\;\epsilon \right)$ denotes the expression (75). Applying Lemma 15 to the second term in the maximum of (76) we obtain (73)–(75). □

**Lemma** **17** (Correct-decoding exponent)**.** *Let $J∼Unif\;\left(\{1,2,\ldots ,\;M\}\right)$ be a random variable, independent of the channel noise, and let x(J)→Y be the random channel-input and channel-output vectors, respectively. Then for any $\tilde{\epsilon },\;\epsilon >0$ and $\;{\tilde{\sigma }}^{2}\geq \sigma^{2}$ there exists $n_{0}=n_{0}\left(\alpha ,\;\beta ,\;s^{2},\;{\tilde{\sigma }}^{2},\;\tilde{\epsilon },\;\epsilon \right)\in N$, such that for any $n>n_{0}$*

(80) $$-\frac{1}{n}\log_{b}\mathrm{Pr}\left\{g(Y)=J\right\}\geq \min\left\{E_{n}\left(R,\;\tilde{\sigma },\;\tilde{\epsilon },\;\epsilon \right),\;E\left(\tilde{\sigma }\right)\right\}\;+\;o\left(1\right),$$

(81) $$E_{n}\left(R,\;\tilde{\sigma },\;\tilde{\epsilon },\;\epsilon \right)≜\min_{\begin{array}{c} P_{X|}:\\ P_{X}\;\in \;P_{n}\left(X_{n}\right),\\ E\left[X^{2}\right]\;\leq \;s^{2}\;+\;\epsilon \end{array}}E_{n}\left(P_{X},\;R,\;\tilde{\sigma },\;\tilde{\epsilon }\;\right)$$

*where $E(\tilde{\sigma })$ and $E_{n}\left(P_{X},\;R,\;\tilde{\sigma },\;\tilde{\epsilon }\;\right)$ are as defined in (74) and (75), respectively, and o(1)→0, as n→∞, depending only on the parameters α, β, $s^{2}+\epsilon$, ${\tilde{\sigma }}^{2}+\tilde{\epsilon }$, and $\sigma^{2}$.*

**Proof.**  Similarly as in [7] [Lemma 5]:

$$\begin{array}{cc} \mathrm{Pr}\left\{g(Y)=J\right\}\;=\;\frac{1}{M}\sum_{\begin{array}{c} P_{X}\;\in \;P_{n}\left(X_{n}\right) \end{array}} & \sum_{\begin{array}{c} 1\;\leq \;j\;\leq \;M\;:\\ x^{q}\left(j\right)\;\in \;T\left(P_{X}\right) \end{array}}\mathrm{Pr}\left\{Y\in D_{J}\;|\;J=j\right\} \end{array}$$

(82) $$\begin{array}{cc} \overset{a}{\leq }\;|\;P_{n}\left(X_{n},\;s^{2}+\epsilon \right)\;|\cdot \frac{1}{M}\max_{\begin{array}{c} P_{X}\;\in \;P_{n}\left(X_{n}\right) \end{array}}\; & \sum_{\begin{array}{c} 1\;\leq \;j\;\leq \;M\;:\\ x^{q}\left(j\right)\;\in \;T\left(P_{X}\right) \end{array}}\mathrm{Pr}\left\{Y\in D_{J}\;|\;J=j\right\} \end{array}$$

(83) $$\begin{array}{cc} \overset{b}{\leq }\;b^{\;n\;\cdot \;o(1)}\;\frac{1}{M}\; & \sum_{\begin{array}{c} 1\;\leq \;j\;\leq \;M\;:\\ x^{q}\left(j\right)\;\in \;T\left(P_{X}^{*}\right) \end{array}}\mathrm{Pr}\left\{Y\in D_{J}\;|\;J=j\right\}\;\overset{c}{=}\;b^{\;n\;\cdot \;o(1)}\mathrm{Pr}\left\{\tilde{g}\left(\tilde{Y}\right)=J\right\}, \end{array}$$

where:

(*a*) holds for $n>n_{0}\left(\alpha ,s^{2},\epsilon \right)$ of Lemma 12;

(*b*) follows by Lemma 7 with $c_{X}=s^{2}+\epsilon$, while $P_{X}^{*}\in P_{n}\left(X_{n}\right)$ is a maximizer of (83);

(*c*) holds for the channel input/output random vectors $\tilde{x}\left(J\right)\to \tilde{Y}$ and a code $\left(\tilde{C},\tilde{g}\right)$, such that $\tilde{x}\left(j\right)=x\left(m_{j}\right)$ with ${\tilde{D}}_{j}=D_{m_{j}}$ for $1\leq j\leq M(P_{X}^{*})$, and ${\tilde{x}}^{q}\left(j\right)\in T\left(P_{X}^{*}\right)$ with ${\tilde{D}}_{j}=⌀$ for $M(P_{X}^{*})<j\leq M$, where $m_{1}<\ldots <m_{M(P_{X}^{*})}$ are the indices of the codewords in the original codebook C with their quantized versions in $T(P_{X}^{*})$. Since all the codewords of $\tilde{C}$ have their quantized versions in $T(P_{X}^{*})$, we can apply Lemma 16 with $c_{X}=s^{2}+\epsilon$ for the RHS of (83) to obtain (80) and (81). □

## 9. PDF to Type

Lemmas 19 and 20 of this section relate between minimums over types and over PDF’s. The next Lemma 18, which has a laborious proof, is required only in the proof of Lemma 19, used for Theorem 1.

**Lemma** **18** (Quantization of PDF)**.** *Let $X_{n}$ be an alphabet defined as in (9), (7) with $\alpha \in \left(0,\frac{1}{4}\right)$. Let $P_{X}\in P_{n}\left(X_{n}\right)$ be a type and $p_{Y|X}\left(\;\cdot \;|\;x\right)\in L$, $\forall x\in X_{n}\;$, be a collection of functions from (12), such that $E_{P_{X}}\left[X^{2}\right]\leq c_{X}$, $E_{P_{X}p_{Y|X}}\left[Y^{2}\right]\leq c_{Y}$, and $E_{P_{X}p_{Y|X}}\left[{\left(Y-X\right)}^{2}\right]\leq c_{XY}$. Then for any alphabet $Y_{n}$ defined as in (9), (7) with $\frac{1}{3}+\frac{2}{3}\alpha <\beta <\frac{2}{3}-\frac{2}{3}\alpha$, there exists a joint type $P_{XY}\in P_{n}\left(X_{n}\times Y_{n}\right)$ with the marginal type $P_{X}$, such that*

(84) $$\begin{array}{cc} \sum_{x\;\in \;X_{n}}P_{X}\left(x\right)\int_{R}p_{Y|X}\left(y\;|\;x\right)\log_{b}\;p_{Y|X}\left(y\;|\;x\right)dy\;\; & \geq \;\;\sum_{\begin{array}{c} x\;\in \;X_{n}\\ y\;\in \;Y_{n} \end{array}}P_{XY}\left(x,y\right)\log_{b}\;\frac{P_{Y|X}\left(y\;|\;x\right)}{\Delta_{\beta ,n}}\;+\;o\left(1\right), \end{array}$$

(85) $$\begin{array}{cc} \sum_{x\;\in \;X_{n}}P_{X}\left(x\right)\int_{R}p_{Y|X}\left(y\;|\;x\right){\left(y-x\right)}^{2}dy\;\; & \geq \;\;\sum_{\begin{array}{c} x\;\in \;X_{n}\\ y\;\in \;Y_{n} \end{array}}P_{XY}\left(x,y\right){\left(y-x\right)}^{2}\;+\;o\left(1\right), \end{array}$$

(86) $$\begin{array}{cc} \int_{R}p_{Y}\left(y\right)\log_{b}\;p_{Y}\left(y\right)dy\;\; & \leq \;\;\sum_{y\;\in \;Y_{n}}P_{Y}\left(y\right)\log_{b}\;\frac{P_{Y}\left(y\right)}{\Delta_{\beta ,n}}\;+\;o\left(1\right), \end{array}$$

*where $p_{Y}\left(y\right)=\sum_{\;x\;\in \;X_{n}}P_{X}\left(x\right)p_{Y|X}\left(y\;|\;x\right)$, ∀y∈R, and o(1)→0, as n→∞, and depends only on the parameters α, β, $c_{X}$, $c_{Y}$, $c_{XY}$, and $\sigma^{2}$ (through (12)).*

The proof is given in the Appendix B.

**Lemma** **19** (PDF to type)**.** *Let $X_{n}$ and $Y_{n}$ be two alphabets defined as in (9), (7) with $\alpha \in \left(0,\frac{1}{4}\right)$ and $\frac{1}{3}+\frac{2}{3}\alpha <\beta <\frac{2}{3}-\frac{2}{3}\alpha$. Then for any $c_{X}$, $c_{XY}$, and ϵ>0 there exists $n_{0}=n_{0}\left(\alpha ,\;\beta ,\;c_{X},\;c_{XY},\;\sigma^{2},\;\epsilon \right)\in N$, such that for any $n>n_{0}$ and for any type $P_{X}\in P_{n}\left(X_{n}\right)$ with $E_{P_{X}}\left[X^{2}\right]\leq c_{X}$:*

(87) $$\underset{\begin{array}{c} p_{Y|X}:\\ p_{Y|X}\left(\;\cdot \;\;|\;x\right)\;\in \;L,\;\forall x,\\ E_{P_{X}p_{Y|X}}\left[{\left(Y-X\right)}^{2}\right]\;\leq \;c_{XY},\\ I(P_{X},\;\;p_{Y|X})\;\leq \;R\;-\;2\epsilon \end{array}}{\inf}\left\{D\left(\;p_{Y|X}\;∥\;\;w\;\;|\;P_{X}\right)\right\}\;\;\geq \min_{\begin{array}{c} P_{Y|X}:\\ P_{XY}\;\in \;P_{n}\left(X_{n}\;\times \;Y_{n}\right),\\ E_{P_{XY}}\left[{\left(Y-X\right)}^{2}\right]\;\leq \;c_{XY}\;+\;\epsilon ,\\ I(P_{X},\;P_{Y|X})\;\leq \;R\;-\;\epsilon \end{array}}\left\{D\left(P_{Y|X}\;∥\;W_{n}\;|\;P_{X}\right)\right\}\;+\;o\left(1\right),$$

*where o(1)→0, as n→∞, and depends only on the parameters α, β, $c_{X}$, $c_{XY}$, and $\sigma^{2}$.*

**Proof.**  For a type $P_{X}$ with a collection of $p_{Y|X}$ such that $E_{P_{X}}\left[X^{2}\right]\leq c_{X}$ and $E_{P_{X}p_{Y|X}}\left[{\left(Y-X\right)}^{2}\right]\leq c_{XY}$, we can find also an upper bound $c_{Y}$ on $E_{P_{X}p_{Y|X}}\left[Y^{2}\right]$. For example, using the Cauchy–Schwarz inequality:

$$E_{P_{X}p_{Y|X}}\left[Y^{2}\right]\;\;\leq \;\;{\left(E_{P_{X}}^{1/2}\left[X^{2}\right]\;+\;E_{P_{X}p_{Y|X}}^{1/2}\left[{\left(Y-X\right)}^{2}\right]\right)}^{2}\;\;\leq \;\;{\left(\sqrt{c_{X}}\;+\;\sqrt{c_{XY}}\right)}^{2}\;\;≜\;\;c_{Y}.$$

Then by Lemma 18 there exists a joint type $P_{XY}$ with the marginal type $P_{X}$, such that simultaneously the three inequalities (84)–(86) are satisfied and it also follows by (10) and (85) that

(88) $$-E_{P_{X}p_{Y|X}}\left[\log_{b}\;w\left(Y\;|\;X\right)\right]\;\;\geq \;\;-E_{P_{XY}}\left[\log_{b}\;W_{n}\left(Y\;|\;X\right)\right]\;+\;\log_{b}\;\Delta_{\beta ,n}\;+\;o\left(1\right).$$

Then the sum of (84) and (88) gives

(89) $$D\left(\;p_{Y|X}\;∥\;\;w\;\;|\;P_{X}\right)\;\;\geq \;\;D\left(P_{Y|X}\;∥\;W_{n}\;|\;P_{X}\right)\;+\;o\left(1\right),$$

while the difference of (84) and (86) gives

(90) $$I\left(P_{X},P_{Y|X}\right)\;\;\leq \;\;I\left(P_{X},\;p_{Y|X}\right)\;+\;o\left(1\right).$$

Note that all o(1) in the above relations are independent of the joint type $P_{XY}$ and the functions $p_{Y|X}$. Therefore by (89), (90) and (85) we conclude, that given any ϵ>0 for *n* sufficiently large for every type $P_{X}$ with the prerequisites of this lemma and every collection of $p_{Y|X}$ that satisfy the conditions under the infimum on the LHS of (87) there exists a joint type $P_{XY}$ such that simultaneously

$$\begin{array}{cc} I\left(P_{X},P_{Y|X}\right)\;\; & \leq \;\;I\left(P_{X},\;p_{Y|X}\right)\;+\;\epsilon ,\\ E_{P_{XY}}\left[{\left(Y-X\right)}^{2}\right]\;\; & \leq \;\;E_{P_{X}p_{Y|X}}\left[{\left(Y-X\right)}^{2}\right]\;+\;\epsilon , \end{array}$$

and (89) holds with a uniform o(1), i.e., independent of $P_{XY}$ and $p_{Y|X}$. It follows that such $P_{XY}$ satisfies also the conditions under the *minimum* on the RHS of (87) and results in the objective function of (87) satisfying (89) with the uniform o(1). Then the minimum itself, which can only possibly be taken over a greater variety of $P_{XY}$, satisfies the inequality (87). □

**Lemma** **20** (Type to PDF)**.** *For any $c_{XY}$ and ϵ>0 there exists $n_{0}=n_{0}\left(\beta ,\;c_{XY},\;\epsilon \right)\in N$, such that for any $n>n_{0}$ and for any type $P_{X}\in P_{n}\left(X_{n}\right)$:*

(91) $$\begin{array}{cc} \min_{\begin{array}{c} P_{Y|X}:\\ P_{XY}\;\in \;P_{n}\left(X_{n}\;\times \;Y_{n}\right),\\ E_{P_{XY}}\left[{\left(Y-X\right)}^{2}\right]\;\leq \;c_{XY} \end{array}}\;\;\;\;\; & \left\{D\left(P_{Y|X}\;∥\;W_{n}\;|\;P_{X}\right)\;+\;|\;R\;-\;I\left(P_{X},P_{Y|X}\right)\;{|}^{+}\right\}\\ \geq \;\;\underset{\begin{array}{c} p_{Y|X}:\\ p_{Y|X}\left(\;\cdot \;\;|\;x\right)\;\in \;F_{n},\;\forall x,\\ E_{P_{X}p_{Y|X}}\left[{\left(Y-X\right)}^{2}\right]\;\leq \;c_{XY}\;+\;\epsilon \end{array}}{\inf}\; & \left\{D\left(\;p_{Y|X}\;∥\;\;w\;\;|\;P_{X}\right)\;+\;|\;R\;-\;I\left(P_{X},\;p_{Y|X}\right)\;{|}^{+}\right\}\;+\;o\left(1\right), \end{array}$$

*where o(1)→0, as n→∞, and depends only on the parameters β and $c_{XY}$.*

**Proof.**  Observe first that any collection of conditional PDF’s $p_{Y|X}$ that satisfies the conditions under the infimum of (91) has finite differential entropies and well-defined quantities $D\left(\;p_{Y|X}\;∥\;\;w\;\;|\;P_{X}\right)$ and $I\left(P_{X},\;p_{Y|X}\right)$. For any conditional type $P_{Y|X}$ from the LHS of (91) we can define a set of histogram-like conditional PDF’s:

(92) $$\begin{array}{cc} p_{Y|X}\left(y\;|\;x\right)\; & ≜\;\frac{P_{Y|X}\left(j\Delta_{\beta ,n}\;|\;x\right)}{\Delta_{\beta ,n}},\;\;\;\;\;\forall y\in \left[\left(j-1/2\right)\Delta_{\beta ,n},\;\left(j+1/2\right)\Delta_{\beta ,n}\right),\;\forall j\in Z,\;\forall x\in S\left(P_{X}\right), \end{array}$$

which are step functions of y∈R for each $x\in S(P_{X})$. Then $I\left(P_{X},\;p_{Y|X}\right)=I\left(P_{X},P_{Y|X}\right)$, and $p_{Y|X}\left(\;\cdot \;|\;x\right)\in F_{n}$, as defined in (11). Analogously to (56)–(58), it can be obtained that

$$E_{P_{X}p_{Y|X}}\left[{\left(Y-X\right)}^{2}\right]\;\;\leq \;\;E_{P_{XY}}\left[{\left(Y-X\right)}^{2}\right]\;+\;\Delta_{\beta ,\;n}\sqrt{c_{XY}}\;+\;\Delta_{\beta ,\;n}^{2}/4.$$

Then also $D\left(\;p_{Y|X}\;∥\;\;w\;\;|\;P_{X}\right)\leq D\left(P_{Y|X}\;∥\;W_{n}\;|\;P_{X}\right)+o\left(1\right)$. Then the lemma follows. □

We use Lemmas 19 and 20 in Section 4 in the derivation of Theorems 1 and 2, respectively.

## Data Availability

The original contributions presented in this study are included in the article. Further inquiries can be directed to the corresponding authors.

## Abbreviations

The following abbreviations are used in this manuscript:

AWGN	Additive white Gaussian noise
PDF	Probability density function
CF	Characteristic function
CDF	Cumulative distribution function
RHS	Right-hand side
LHS	Left-hand side

## Appendix A

**Proof** **of Lemma 3.** Adding the two power constraints together, we successively obtain the following inequalities:

$$\begin{array}{cc} \sum_{\left(x,\;y\right)\;\in \;S\left(P_{XY}\right)}P_{XY}\left(x,y\right)\left(x^{2}+y^{2}\right)\;\; & \leq \;\;c_{X}+c_{Y},\\ \sum_{\left(x,\;y\right)\;\in \;S\left(P_{XY}\right)}\frac{1}{n}\left(x^{2}+y^{2}\right)\;\; & \leq \;\;c_{X}+c_{Y},\\ \sum_{\left(x,\;y\right)\;\in \;S\left(P_{XY}\right)}\frac{1}{\left|\;S(P_{XY})\;\right|}\left(x^{2}+y^{2}\right)\;\; & \leq \;\;\frac{n(c_{X}+c_{Y})}{\left|\;S(P_{XY})\;\right|},\\ E\left[{∥U∥}^{2}\right]\;\; & \leq \;\;\frac{n(c_{X}+c_{Y})}{\left|\;S(P_{XY})\;\right|}, \end{array}$$

where $U∼\mathrm{Discrete}\text{-}\mathrm{Uniform}\left(S(P_{XY})\right)$. To this let us add a *continuously* distributed random vector

$$D\;∼\;\mathrm{Continuous}\text{-}\mathrm{Uniform}\left(\;\left[-\Delta_{\alpha ,\;n}/2,\;\Delta_{\alpha ,\;n}/2\right)\;\times \;\left[-\Delta_{\beta ,\;n}/2,\;\Delta_{\beta ,\;n}/2\right)\;\right),$$

independent of U. Then $\tilde{U}\;=\;U+D\;∼\;\mathrm{Continuous}\text{-}\mathrm{Uniform}\left(A\right)$, where

$$A\;\;≜\;\;\underset{\left(x,\;y\right)\;\in \;S\left(P_{XY}\right)}{⋃}\left\{x\;+\;[-\Delta_{\alpha ,\;n}/2,\;\Delta_{\alpha ,\;n}/2)\right\}\;\times \;\left\{y\;+\;[-\Delta_{\beta ,\;n}/2,\;\Delta_{\beta ,\;n}/2)\right\}.$$

So we have

(A1) $$\begin{array}{cc} \frac{n(c_{X}+c_{Y})}{\left|\;S(P_{XY})\;\right|}\;+\;\frac{\Delta_{\alpha ,n}^{2}+\Delta_{\beta ,n}^{2}}{12}\;\; & \geq \;\;E\left[{∥U∥}^{2}\right]\;+\;E\left[{∥D∥}^{2}\right]\;\;=\;\;E\left[∥\tilde{U}{∥}^{2}\right]\;\;=\;\;\frac{1}{\mathrm{vol}(A)}\int_{A}{∥\tilde{u}∥}^{2}d^{2}\tilde{u}\\ \; & =\;\;\frac{1}{\mathrm{vol}(A)}\left[\int_{A\;\cap \;B}∥\tilde{u}{∥}^{2}d^{2}\tilde{u}\;+\int_{A\;\cap \;B^{c}}{∥\tilde{u}∥}^{2}d^{2}\tilde{u}\right]\\ \; & \overset{a}{\geq }\;\;\frac{1}{\mathrm{vol}(A)}\left[\int_{A\;\cap \;B}∥\tilde{u}{∥}^{2}d^{2}\tilde{u}\;+\int_{A^{c}\;\cap \;B}{∥t∥}^{2}d^{2}t\right]\\ \; & =\;\;\frac{1}{\mathrm{vol}(A)}\int_{B}{∥t∥}^{2}d^{2}t, \end{array}$$

where for (*a*) we use the disk set $B\;≜\;\left\{{t:\;\pi ∥t∥}^{2}\;\leq \;\mathrm{vol}\left(A\right)\right\}$, centered around zero and of the same total area as *A*, and the resulting property

$$\begin{array}{cc} \mathrm{vol}\left(A\cap B\right)\;+\;\mathrm{vol}\left(A\cap B^{c}\right)\;\;=\;\;\mathrm{vol}\left(A\right)\;\; & =\;\;\mathrm{vol}\left(B\right)\;\;=\;\;\mathrm{vol}\left(A\cap B\right)\;+\;\mathrm{vol}\left(A^{c}\cap B\right),\\ \mathrm{vol}(A\cap B^{c})\;\; & =\;\;\mathrm{vol}(A^{c}\cap B),\\ \int_{A\;\cap \;B^{c}}{∥\tilde{u}∥}^{2}d^{2}\tilde{u}\;\;\geq \;\;\frac{\mathrm{vol}(A)}{\pi }\int_{A\;\cap \;B^{c}}d^{2}\tilde{u}\;\; & =\;\;\frac{\mathrm{vol}(A)}{\pi }\int_{A^{c}\;\cap \;B}d^{2}t\;\;\geq \;\;\int_{A^{c}\;\cap \;B}{∥t∥}^{2}d^{2}t. \end{array}$$

Integrating on the RHS of (A1), we obtain

$$\begin{array}{cc} \frac{\mathrm{vol}(A)}{2\pi }\;\;=\;\;\frac{|\;S\left(P_{XY}\right)\;|\;\cdot \;\Delta_{\alpha ,n}\Delta_{\beta ,n}}{2\pi }\;\; & \leq \;\;\frac{n(c_{X}+c_{Y})}{\left|\;S(P_{XY})\;\right|}\;+\;\frac{\Delta_{\alpha ,n}^{2}+\Delta_{\beta ,n}^{2}}{12},\\ \frac{|\;S\left(P_{XY}\right){\;|}^{\;2}\cdot \Delta_{\alpha ,n}\Delta_{\beta ,n}}{2\pi n}\;\; & \leq \;\;c_{X}+c_{Y}\;+\;\frac{\Delta_{\alpha ,n}^{2}+\Delta_{\beta ,n}^{2}}{12}\cdot \underset{\leq \;1}{\underset{︸}{\frac{\left|\;S(P_{XY})\;\right|}{n}}}\;\;\leq \;\;c_{X}+c_{Y}+1/6,\\ |\;S\left(P_{XY}\right){\;|}^{\;2}\;\; & \leq \;\;2\pi \left(c_{X}+c_{Y}+1/6\right)\cdot n\Delta_{\alpha ,n}^{-1}\Delta_{\beta ,n}^{-1}\\ \; & =\;\;2\pi \left(c_{X}+c_{Y}+1/6\right)\cdot n^{1\;+\;\alpha \;+\;\beta }. \end{array}$$

□

**Proof** **of Lemma 4.** Similarly as in Lemma 3, the power constraint gives the following succession of inequalities:

$$\begin{array}{cc} \sum_{x\;\in \;S(P_{X})}P_{X}\left(x\right)x^{2}\;\; & \leq \;\;c_{X},\\ \sum_{x\;\in \;S(P_{X})}\frac{1}{n}x^{2}\;\; & \leq \;\;c_{X},\\ \sum_{x\;\in \;S(P_{X})}\frac{1}{\left|\;S(P_{X})\;\right|}x^{2}\;\; & \leq \;\;\frac{n}{\left|\;S(P_{X})\;\right|}c_{X},\\ E\left[U^{2}\right]\;\; & \leq \;\;\frac{n}{\left|\;S(P_{X})\;\right|}c_{X}, \end{array}$$

where $U∼\mathrm{Discrete}\text{-}\mathrm{Uniform}\left(S(P_{X})\right)$. To this let us add a *continuously* distributed random variable

$$D\;∼\;\mathrm{Continuous}\text{-}\mathrm{Uniform}\left(\;[-\Delta_{\alpha ,\;n}/2,\;\Delta_{\alpha ,\;n}/2)\;\right),$$

independent of *U*. Then $\tilde{U}\;=\;U+D\;∼\;\mathrm{Continuous}\text{-}\mathrm{Uniform}\left(A\right)$, where

$$A\;\;≜\;\;\underset{x\;\in \;S(P_{X})}{⋃}\left\{x+[-\Delta_{\alpha ,\;n}/2,\;\Delta_{\alpha ,\;n}/2)\right\}.$$

So we have

(A2) $$\begin{array}{cc} \frac{n}{\left|\;S(P_{X})\;\right|}c_{X}\;+\;\frac{\Delta_{\alpha ,\;n}^{2}}{12}\;\;\geq \;\;E\left[U^{2}\right]\;+\;E\left[D^{2}\right]\;\;=\;\;E\left[{\tilde{U}}^{2}\right]\;\; & =\;\;\frac{1}{\mathrm{vol}(A)}\int_{A}{\tilde{u}}^{\;2}d\tilde{u}\\ \; & =\;\;\frac{1}{\mathrm{vol}(A)}\left[\int_{A\;\cap \;B}{\tilde{u}}^{\;2}d\tilde{u}\;+\int_{A\;\cap \;B^{c}}{\tilde{u}}^{\;2}d\tilde{u}\right]\\ \; & \overset{a}{\geq }\;\;\frac{1}{\mathrm{vol}(A)}\left[\int_{A\;\cap \;B}{\tilde{u}}^{\;2}d\tilde{u}\;+\int_{A^{c}\;\cap \;B}t^{\;2}dt\right]\\ \; & =\;\;\frac{1}{\mathrm{vol}(A)}\int_{B}t^{\;2}dt, \end{array}$$

where for (*a*) we use the interval set $B\;≜\;\left[-\mathrm{vol}(A)/2,\;\mathrm{vol}(A)/2\;\right]$, centered around zero and of the same total length as *A* with the resulting property that $\mathrm{vol}\left(A\cap B^{c}\right)=\mathrm{vol}\left(A^{c}\cap B\right)$, so that

$$\int_{A\;\cap \;B^{c}}{\tilde{u}}^{\;2}d\tilde{u}\;\;\geq \;\;\frac{{\left(\mathrm{vol}\left(A\right)\right)}^{2}}{4}\int_{A\;\cap \;B^{c}}d\tilde{u}\;\;=\;\;\frac{{\left(\mathrm{vol}\left(A\right)\right)}^{2}}{4}\int_{A^{c}\;\cap \;B}dt\;\;\geq \;\;\int_{A^{c}\;\cap \;B}t^{\;2}dt.$$

Integrating on the RHS of (A2), we obtain

$$\begin{array}{cc} \frac{{\left(\mathrm{vol}\left(A\right)\right)}^{2}}{12}\;\;=\;\;\frac{(|\;S\left(P_{X}\right)\;{\left|\;\cdot \;\Delta_{\alpha ,n}\right)}^{2}}{12}\;\; & \leq \;\;\frac{n}{\left|\;S(P_{X})\;\right|}c_{X}\;+\;\frac{\Delta_{\alpha ,n}^{2}}{12},\\ |\;S\left(P_{X}\right){\;|}^{\;3}\;\; & \leq \;\;12c_{X}n\Delta_{\alpha ,n}^{-2}\;+\;\left|\;S\left(P_{X}\right)\;\right|\\ \; & =\;\;12c_{X}n^{1\;+\;2\alpha }\;+\;\underset{\leq \;n}{\underset{︸}{\left|\;S(P_{X})\;\right|}}\;\;\leq \;\;\left(12c_{X}+1\right)n^{1\;+\;2\alpha }. \end{array}$$

□

## Appendix B

**Proof** **of Lemma 18.** Using $P_{X}$ and $p_{Y|X}$, it is convenient to define a joint probability density function over $R^{2}$ as

(A3) $$\begin{array}{lll} p_{XY}\left(x,y\right) & ≜ & \frac{P_{X}\left(i\Delta_{\alpha ,\;n}\right)}{\Delta_{\alpha ,\;n}}p_{Y|X}\left(y\;|\;i\Delta_{\alpha ,\;n}\right),\\ & & \;\forall x\in \left[\left(i-1/2\right)\Delta_{\alpha ,n},\;\left(i+1/2\right)\Delta_{\alpha ,n}\right),\;\forall i\in Z,\;\forall y\in R, \end{array}$$

which is changing only stepwise in *x*-direction. Note that $p_{Y}$ of (86) is the *y*-marginal of $p_{XY}$. This gives

(A4) $$E_{p_{XY}}\left[X^{2}\right]\;=\;E_{P_{X}}\left[X^{2}\right]\;+\;\frac{\Delta_{\alpha ,\;n}^{2}}{12},\;\;\;\;\;\;E_{p_{XY}}\left[Y^{2}\right]\;=\;E_{P_{X}p_{Y|X}}\left[Y^{2}\right].$$

We proceed in two stages. First we quantize $p_{XY}\left(x,y\right)$ by rounding it *down* and check the effect of this on the LHS of (84)–(86). Then we complement the total probability back to 1, so that the type $P_{X}$ is conserved, and check the effect of this on the RHS of (84)–(86). The quantization of $p_{XY}\left(x,y\right)$ is done by first replacing it with its infimum in each rectangle

$$\left[\left(i-1/2\right)\Delta_{\alpha ,n},\;\left(i+1/2\right)\Delta_{\alpha ,n}\right)\;\times \;\left[\left(j-1/2\right)\Delta_{\beta ,n},\;\left(j+1/2\right)\Delta_{\beta ,n}\right):$$

(A5) $$\begin{array}{cc} p_{XY}^{\inf}\left(x,y\right)\;\; & ≜\;\;\underset{\left(j\;-\;1/2\right)\Delta_{\beta ,n}\;\leq \;\tilde{y}\;<\;\left(j\;+\;1/2\right)\Delta_{\beta ,n}}{\inf}p_{XY}\left(x,\tilde{y}\right),\\ \; & \;\;\;\;\;\;\;\;\;\;\;\;\;\;\;\;\;\;\;\;\;\;\;\;\forall y\in \left[\left(j-1/2\right)\Delta_{\beta ,\;n},\;\left(j+1/2\right)\Delta_{\beta ,\;n}\right),\;\forall j\in Z,\;\forall x\in R, \end{array}$$

and then quantizing this infimum down to the nearest value $k\Delta_{\gamma ,n}$, k=0,1,2,…:

(A6) $$\begin{array}{cc} p_{XY}^{q}\left(x,y\right)\;\; & ≜\;\;\left\lfloor p_{XY}^{\inf}\left(x,y\right)/\Delta_{\gamma ,n}\right\rfloor \cdot \Delta_{\gamma ,n},\;\;\;\;\;\;\forall \left(x,y\right)\in R^{2}. \end{array}$$

Due to (8), the integral of $p_{XY}^{q}$ over $R^{2}$ can be smaller than 1 only by an integer multiple of 1/n. The resulting difference from $p_{XY}\left(x,y\right)$ at each point $\left(x,y\right)\in R^{2}$ can be bounded by a sum of two terms as

(A7) $$\begin{array}{cc} 0\;\;\leq \;\;p_{XY}\left(x,y\right)\;-\;p_{XY}^{q}\left(x,y\right)\;\; & \leq \;\;\underset{\mathrm{minimization}}{\underset{︸}{\left(K/\Delta_{\alpha ,n}\right)\cdot \Delta_{\beta ,n}}}\;+\underset{\mathrm{quantization}}{\underset{︸}{\Delta_{\gamma ,n}}}\\ \; & =\;\;Kn^{\alpha \;-\;\beta }\;+\;n^{\alpha \;+\;\beta \;-\;1}\;\;\leq \;\;\left(K+1\right)n^{-\delta }\;\;≜\;\;h, \end{array}$$

where δ≜min{β,1−β}−α and *K* is the parameter from (12). For (86) we will require the *y*-marginal of $p_{XY}^{q}$ from (A6), defined in the usual manner:

(A8) $$\begin{array}{cc} p_{Y}^{q}\left(y\right)\;\;≜\;\;\int_{R}p_{XY}^{q}\left(x,y\right)dx\;\; & \overset{a}{=}\;\;\sum_{x\;\in \;S(P_{X})}p_{XY}^{q}\left(x,y\right)\Delta_{\alpha ,n}\;\;\overset{b}{\geq }\;\;\sum_{x\;\in \;S(P_{X})}\left(p_{XY}\left(x,y\right)-h\right)\Delta_{\alpha ,n}\\ \; & =\;\;\sum_{x\;\in \;S(P_{X})}p_{XY}\left(x,y\right)\Delta_{\alpha ,n}\;-\;h\sum_{x\;\in \;S(P_{X})}\Delta_{\alpha ,n}\\ \; & =\;\;p_{Y}\left(y\right)\;-\;h\;\;\left|\;S\left(P_{X}\right)\;\right|\;\;\Delta_{\alpha ,n},\;\;\;\;\;\;\forall y\in R, \end{array}$$

where the equality (*a*) follows from (A3), (A5) and (A6), and the inequality (*b*) follows by (A7). Then

(A9) $$\begin{array}{cc} 0\;\;\leq \;\;p_{Y}\left(y\right)\;-\;p_{Y}^{q}\left(y\right)\;\; & \leq \;\;h\;\;|\;S\left(P_{X}\right)\;|\;\;\Delta_{\alpha ,n}\;\;\leq \;\;\left(K+1\right){\left(12c_{X}+1\right)}^{1/3}n^{-\delta_{1}}\;\;≜\;\;\tilde{h}, \end{array}$$

where the last inequality follows by Lemma 4, (A7), (7), with $\delta_{1}\;≜\;\min\;\left\{\beta ,\;1-\beta \right\}\;-\;\left(1+2\alpha \right)/3$. Note that the previously defined $\delta >\delta_{1}$, while $\delta_{1}>0$ if and only if $\alpha \in \left(0,\frac{1}{4}\right)$ and $\frac{1}{3}+\frac{2}{3}\alpha <\beta <\frac{2}{3}-\frac{2}{3}\alpha$.
*The LHS of (84)*
Now consider the LHS of (84). Note that each function $p_{Y|X}\left(\;\cdot \;|\;x\right)$ in (84) is bounded and has a finite variance. It follows that it has a finite differential entropy. With (A3) we can rewrite the LHS of (84) as

(A10) $$\begin{array}{cc} \sum_{x\;\in \;X_{n}}P_{X}\left(x\right)\int_{R}p_{Y|X}\left(y\;|\;x\right)\log_{b}\;p_{Y|X}\left(y\;|\;x\right)dy\;\;=\;\; & \int \int_{R^{2}}p_{XY}\left(x,y\right)\log_{b}\;p_{XY}\left(x,y\right)dxdy\\ \; & -\sum_{x\;\in \;X_{n}}P_{X}\left(x\right)\log_{b}\;\frac{P_{X}\left(x\right)}{\Delta_{\alpha ,\;n}}. \end{array}$$

Let us examine the possible increase in (A10) when $p_{XY}$ is replaced with $p_{XY}^{q}$ defined by (A5) and (A6). For this, let us define a set in $R^{2}$ with respect to the parameter *h* of (A7):

(A11) $$\begin{array}{cc} A\;\; & ≜\;\;\left\{\left(x,y\right):\;\;p_{XY}\left(x,y\right)>h\right\}, \end{array}$$

which is a countable union of disjoint rectangles by the definition of $p_{XY}$ in (A3). Then

(A12) $$\begin{array}{cc} \; & \;\;\;\;\int \int_{R^{2}}p_{XY}^{q}\left(x,y\right)\log_{b}\;p_{XY}^{q}\left(x,y\right)dxdy\;-\;\int \int_{R^{2}}p_{XY}\left(x,y\right)\log_{b}\;p_{XY}\left(x,y\right)dxdy\\ = & \;\;\left[\int \int_{A^{c}}p_{XY}^{q}\left(x,y\right)\log_{b}\;p_{XY}^{q}\left(x,y\right)dxdy\;-\;\int \int_{A^{c}}p_{XY}\left(x,y\right)\log_{b}\;p_{XY}\left(x,y\right)dxdy\right]\\ \; & +\int \int_{A}\left[\;p_{XY}^{q}\left(x,y\right)\log_{b}\;p_{XY}^{q}\left(x,y\right)\;-\;p_{XY}\left(x,y\right)\log_{b}\;p_{XY}\left(x,y\right)\right]\;dxdy. \end{array}$$

Note that the minimum of the function $f\left(t\right)=t\;\log_{b}\;t$ occurs at t=1/e. Then for h≤1/e we have $p_{XY}\left(x,y\right)\log_{b}\;p_{XY}\left(x,y\right)\;\leq \;p_{XY}^{q}\left(x,y\right)\log_{b}\;p_{XY}^{q}\left(x,y\right)\;\leq \;0$ for all $\left(x,y\right)\in A^{c}$ and the first of the two terms in (A12) is upper-bounded as

(A13) $$\int \int_{A^{c}}p_{XY}^{q}\left(x,y\right)\log_{b}\;p_{XY}^{q}\left(x,y\right)dxdy\;-\;\int \int_{A^{c}}p_{XY}\left(x,y\right)\log_{b}\;p_{XY}\left(x,y\right)dxdy$$

$$\overset{h\;\leq \;1/e}{\leq }\;\;-\int \int_{A^{c}}p_{XY}\left(x,y\right)\log_{b}\;p_{XY}\left(x,y\right)dxdy\;\;\overset{\left(*\right)}{=}\;\;-p\int \int_{R^{2}}p_{XY}^{c}\left(x,y\right)\log_{b}\;p_{XY}^{c}\left(x,y\right)dxdy\;-\;p\;\log_{b}\;p,$$

where the equality (∗) is appropriate for the case when the upper bound is positive, with the definitions:

(A14) $$p\;\;≜\;\;\int \int_{A^{c}}p_{XY}\left(x,y\right)dxdy,$$

$$p_{XY}^{c}\left(x,y\right)\;\;≜\;\;\{\begin{array}{cc} p_{XY}\left(x,y\right)/p, & \left(x,y\right)\in A^{c},\\ 0, & o.w. \end{array}$$

Next we upper-bound the entropy of the probability density function $p_{XY}^{c}$ on the RHS of (A13) by that of a Gaussian PDF. By (A4) we have

(A15) $$\begin{array}{cc} \; & \;\;\;\;\;\;\;\;\;\;\;\;\;\;\;\;\;\;c_{X}\;+\;1/12\;+\;c_{Y}\;\geq \;\int \int_{A^{c}}p_{XY}\left(x,y\right)\left(x^{2}+y^{2}\right)dxdy\;=\;p\int \int_{R^{2}}p_{XY}^{c}\left(x,y\right)\left(x^{2}+y^{2}\right)dxdy,\\ \; & \;\;\;\;\;\;\;\;\;\;\;\;\;\;\;\;\left(c_{X}\;+\;1/12\;+\;c_{Y}\right)/p\;\geq \;\int_{R}p_{X}^{c}\left(x\right)x^{2}dx\;+\;\int_{R}p_{Y}^{c}\left(y\right)y^{2}dy,\\ \; & \log_{b}\;\left(2\pi e(c_{X}\;+\;1/12\;+\;c_{Y})/p\right)\;\geq \;-\int \int_{R^{2}}p_{XY}^{c}\left(x,y\right)\log_{b}\;p_{XY}^{c}\left(x,y\right)dxdy. \end{array}$$

So we can rewrite the bound of (A13) in terms of *p* defined by (A14):

(A16) $$\begin{array}{cc} \; & \int \int_{A^{c}}p_{XY}^{q}\left(x,y\right)\log_{b}\;p_{XY}^{q}\left(x,y\right)dxdy\;-\;\int \int_{A^{c}}p_{XY}\left(x,y\right)\log_{b}\;p_{XY}\left(x,y\right)dxdy\\ \overset{h\;\leq \;1/e}{\leq }\;\; & -2p\;\log_{b}\;p\;+\;p\;\log_{b}\;\left(2\pi e(c_{X}+c_{Y}+1/12)\right). \end{array}$$

From (A11) and (A14) it is clear that p→0 as h→0. In order to relate between them, let us rewrite the inequality in (A15) again as

$$\begin{array}{cc} \; & c_{X}\;+\;1/12\;+\;c_{Y}\;\;\geq \;\;\int \int_{A^{c}}p_{XY}\left(x,y\right)\left(x^{2}+y^{2}\right)dxdy\;\; \end{array}$$

$$\begin{array}{cc} =\;\; & \int \int_{B_{1}}h\cdot \left(x^{2}+y^{2}\right)dxdy\;+\;\int \int_{A^{c}\;\cap \;B_{1}^{c}}p_{XY}\left(x,y\right)\left(x^{2}+y^{2}\right)dxdy\;-\;\int \int_{A\;\cap \;B_{1}}h\cdot \left(x^{2}+y^{2}\right)dxdy\\ \; & \;\;\;\;\;\;\;\;\;\;\;\;\;\;\;\;\;\;\;\;\;\;\;\;\;\;\;\;\;\;\;\;\;\;\;\;\;\;\;\;\;\;\;\;\;\;\;\;\;\;\;\;\;\;\;\;\;\;\;\;\;\;\;\;\;\;\;\;\;\;\;\;-\int \int_{A^{c}\;\cap \;B_{1}}\left(h-p_{XY}\left(x,y\right)\right)\left(x^{2}+y^{2}\right)dxdy\\ \geq \;\; & \int \int_{B_{1}}h\cdot \left(x^{2}+y^{2}\right)dxdy\;+\;\frac{p}{h\pi }\int \int_{A^{c}\;\cap \;B_{1}^{c}}p_{XY}\left(x,y\right)dxdy\;-\;\frac{p}{h\pi }\int \int_{A\;\cap \;B_{1}}hdxdy\\ \; & \;\;\;\;\;\;\;\;\;\;\;\;\;\;\;\;\;\;\;\;\;\;\;\;\;\;\;\;\;\;\;\;\;\;\;\;\;\;\;\;\;\;\;\;\;\;\;\;\;\;\;\;\;\;\;\;\;\;\;\;\;-\frac{p}{h\pi }\int \int_{A^{c}\;\cap \;B_{1}}\left(h-p_{XY}\left(x,y\right)\right)dxdy\\ =\;\; & \int \int_{B_{1}}h\cdot \left(x^{2}+y^{2}\right)dxdy\;\;=\;\;\frac{p^{2}}{2\pi h}, \end{array}$$

where we use the disk set $B_{1}≜\left\{\left(x,y\right):\;h\pi \left(x^{2}+y^{2}\right)\;\leq \;p\right\}$, centered around zero. This results in the following upper bound on *p* in terms of *h*:

(A17) $$c_{1}h^{1/2}\;\;\geq \;\;p,$$

where $c_{1}≜\sqrt{2\pi (c_{X}+c_{Y}+1/12)}$. Substituting the LHS of (A17) in (A16) in place of *p*, we obtain the following upper bound on the first half of (A12) in terms of *h* of (A7) and (A11):

(A18) $$\begin{array}{cc} \; & \int \int_{A^{c}}p_{XY}^{q}\left(x,y\right)\log_{b}\;p_{XY}^{q}\left(x,y\right)dxdy\;-\;\int \int_{A^{c}}p_{XY}\left(x,y\right)\log_{b}\;p_{XY}\left(x,y\right)dxdy\\ \leq \;\; & c_{1}h^{1/2}\;\log_{b}\;\left(e/h\right),\;\;\;\;\;\;h\;\leq \;1/{\left(c_{1}e\right)}^{2}. \end{array}$$

In the second term of (A12) for (x,y)∈A the integrand can be upper-bounded by Lemma A1 with its parameters *t* and $t_{1}$ such that

$$\begin{array}{cc} t_{1}\;=\;p_{XY}^{q}\left(x,y\right)\; & \leq \;p_{XY}\left(x,y\right)\;=\;t_{1}+t,\;\;\;\;\;\;\;\;\;t\;\leq \;h\;\leq \;1/e. \end{array}$$

This gives

(A19) $$\begin{array}{cc} \; & \int \int_{A}\left[\;p_{XY}^{q}\left(x,y\right)\log_{b}\;p_{XY}^{q}\left(x,y\right)\;-\;p_{XY}\left(x,y\right)\log_{b}\;p_{XY}\left(x,y\right)\right]\;dxdy\\ \;\;\leq \; & \mathrm{vol}\left(A\right)\;h\;\log_{b}\;\left(1/h\right),\;\;\;\;\;\;h\;\leq \;1/e, \end{array}$$

where vol(A) is the total area of *A*. To find an upper bound on vol(A), we use (A4):

$$\begin{array}{cc} c_{X}\;+\;1/12\;+\;c_{Y}\;\; & \geq \;\;\int \int_{A}p_{XY}\left(x,y\right)\left(x^{2}+y^{2}\right)dxdy\;\;\geq \;\;\int \int_{A}h\cdot \left(x^{2}+y^{2}\right)dxdy\\ \; & =\;\;h\left[\int \int_{A\;\cap \;B_{2}}\left(x^{2}+y^{2}\right)dxdy\;+\int \int_{A\;\cap \;B_{2}^{c}}\left(x^{2}+y^{2}\right)dxdy\right]\\ \; & \overset{a}{\geq }\;\;h\left[\int \int_{A\;\cap \;B_{2}}\left(x^{2}+y^{2}\right)dxdy\;+\int \int_{A^{c}\;\cap \;B_{2}}\left(x^{2}+y^{2}\right)dxdy\right]\\ \; & =\;\;\int \int_{B_{2}}h\cdot \left(x^{2}+y^{2}\right)dxdy\;\;=\;\;\frac{h}{2\pi }{\left(\mathrm{vol}\left(A\right)\right)}^{2}, \end{array}$$

where in (*a*) we use the disk set $B_{2}\;≜\;\left\{\left(x,y\right):\;\pi \left(x^{2}+y^{2}\right)\;\leq \;\mathrm{vol}\left(A\right)\right\}$, centered around zero, of the same total area as *A*, and the resulting property that $\mathrm{vol}\left(A^{c}\cap B_{2}\right)=\mathrm{vol}\left(A\cap B_{2}^{c}\right)$. So that

(A20) $$c_{1}h^{-1/2}\;\;\geq \;\;\mathrm{vol}\left(A\right).$$

Continuing (A19), we therefore obtain the following upper bound on the second term in (A12):

(A21) $$\begin{array}{cc} \int \int_{A}\left[\;p_{XY}^{q}\left(x,y\right)\log_{b}\;p_{XY}^{q}\left(x,y\right)\;-\;p_{XY}\left(x,y\right)\log_{b}\;p_{XY}\left(x,y\right)\right]dxdy & \leq \;c_{1}h^{1/2}\;\log_{b}\;\left(1/h\right),\;\;\;h\leq 1/e. \end{array}$$

Putting (A12), (A18) and (A21) together:

(A22) $$\begin{array}{cc} \; & \int \int_{R^{2}}p_{XY}^{q}\left(x,y\right)\log_{b}\;p_{XY}^{q}\left(x,y\right)dxdy\;-\;\int \int_{R^{2}}p_{XY}\left(x,y\right)\log_{b}\;p_{XY}\left(x,y\right)dxdy\\ \leq & 2c_{1}h^{1/2}\;\log_{b}\left(\sqrt{e}/h\right),\;\;\;\;\;\;h\;\leq \;1/{\left(c_{1}e\right)}^{2}, \end{array}$$

where $c_{1}$ and *h* are such as in (A17) and (A7), respectively. So if δ>0 in (A7), then the possible increase in (A10) caused by substitution of $p_{XY}^{q}$ in place of $p_{XY}$ is at most o(1). Later on, for the RHS of (84)–(86) we will require also the loss in the total probability incurred in the replacement of $p_{XY}$ by $p_{XY}^{q}$. This loss is strictly positive and tends to zero with *h* of (A7):

(A23) $$\begin{array}{cc} 0\;\;<\;\;p_{1}\;\; & ≜\;\;\underset{=\;1}{\underset{︸}{\int \int_{R^{2}}p_{XY}\left(x,y\right)dxdy}}\;-\;\int \int_{R^{2}}p_{XY}^{q}\left(x,y\right)dxdy\\ \; & \overset{a}{\leq }\;\;\underset{=\;p}{\underset{︸}{\int \int_{A^{c}}p_{XY}\left(x,y\right)dxdy}}\;+\;\int \int_{A}\underset{\leq \;h}{\underset{︸}{\left(p_{XY}\left(x,y\right)-p_{XY}^{q}\left(x,y\right)\right)}}dxdy\\ \; & \overset{b}{\leq }\;\;p\;+\;h\cdot \mathrm{vol}\left(A\right)\;\;\overset{c}{\leq }\;\;c_{1}h^{1/2}\;+\;c_{1}h^{1/2}\;\;=\;\;2c_{1}h^{1/2}, \end{array}$$

where the set *A* in (*a*) is defined in (A11), (*b*) follows by (A14) and (A7), and (*c*) follows by (A17) and (A20).
*The LHS of (86)*
Consider next the LHS of (86). Since $p_{Y}\in L$ and has a finite variance, its differential entropy is finite. Let us examine the possible decrease in the LHS of (86) when $p_{Y}$ is replaced with $p_{Y}^{q}$ defined in (A8). For this, let us define a set in R with respect to the parameter $\tilde{h}$ of (A9):

(A24) $$\begin{array}{cc} \tilde{A}\;\; & ≜\;\;\left\{y:\;\;p_{Y}\left(y\right)>\tilde{h}\right\}, \end{array}$$

which is a countable union of disjoint open intervals. Then

(A25) $$\begin{array}{cc} \; & \int_{R}p_{Y}\left(y\right)\log_{b}\;p_{Y}\left(y\right)dy\;-\;\int_{R}p_{Y}^{q}\left(y\right)\log_{b}\;p_{Y}^{q}\left(y\right)dy\\ =\; & \int_{{\tilde{A}}^{c}}\left[\;p_{Y}\left(y\right)\log_{b}\;p_{Y}\left(y\right)\;-\;p_{Y}^{q}\left(y\right)\log_{b}\;p_{Y}^{q}\left(y\right)\right]\;dy\;+\;\int_{\tilde{A}}\left[\;p_{Y}\left(y\right)\log_{b}\;p_{Y}\left(y\right)\;-\;p_{Y}^{q}\left(y\right)\log_{b}\;p_{Y}^{q}\left(y\right)\right]\;dy. \end{array}$$

For $\tilde{h}\leq 1/e$ we have $p_{Y}\left(y\right)\log_{b}\;p_{Y}\left(y\right)\;\leq \;p_{Y}^{q}\left(y\right)\log_{b}\;p_{Y}^{q}\left(y\right)\;\leq \;0$ for all $y\in {\tilde{A}}^{c}$ and the first of the two terms in (A25) is non-positive:

(A26) $$\begin{array}{cc} \; & \int_{{\tilde{A}}^{c}}\left[\;p_{Y}\left(y\right)\log_{b}\;p_{Y}\left(y\right)\;-\;p_{Y}^{q}\left(y\right)\log_{b}\;p_{Y}^{q}\left(y\right)\right]\;dy\;\;\leq \;\;0. \end{array}$$

In the second term of (A25) for $y\in \tilde{A}$, the integrand can be upper-bounded by Lemma A1 with its parameters *t* and $t_{1}$ such that

$$\begin{array}{cc} t_{1}\; & =\;p_{Y}^{q}\left(y\right)\;\leq \;t_{1}+t\;=\;p_{Y}\left(y\right)\;\leq \;\underset{y\;\in \;R}{\sup}p_{Y}\left(y\right)\;\leq \;\sqrt{K},\;\;\;\;\;\;\;\;\;t\;\leq \;\tilde{h}\;\leq \;1/e, \end{array}$$

where *K* is the parameter from (12). This gives

(A27) $$\begin{array}{cc} \int_{\tilde{A}}\left[\;p_{Y}\left(y\right)\log_{b}\;p_{Y}\left(y\right)\;-\;p_{Y}^{q}\left(y\right)\log_{b}\;p_{Y}^{q}\left(y\right)\right]\;dy\;\;\; & \overset{\tilde{h}\;\leq \;1/e}{\leq }\;\;\mathrm{vol}\left(\tilde{A}\right)\;\tilde{h}\cdot \max\left\{\log_{b}\;\left(1/\tilde{h}\right),\;\log_{b}\left(e\sqrt{K}\right)\right\}, \end{array}$$

where $\mathrm{vol}(\tilde{A})$ is the total length of $\tilde{A}$. It remains to find an upper bound on $\mathrm{vol}(\tilde{A})$. We use (A4):

$$\begin{array}{cc} c_{Y}\;\;\geq \;\;\int_{\tilde{A}}p_{Y}\left(y\right)y^{2}dy\;\;\geq \;\;\int_{\tilde{A}}\tilde{h}\cdot y^{2}dy\;\; & =\;\;\tilde{h}\left[\int_{\tilde{A}\;\cap \;{\tilde{B}}_{2}}y^{2}dy\;+\int_{\tilde{A}\;\cap \;{\tilde{B}}_{2}^{c}}y^{2}dy\right]\\ \; & \overset{a}{\geq }\;\;\tilde{h}\left[\int_{\tilde{A}\;\cap \;{\tilde{B}}_{2}}y^{2}dy\;+\int_{{\tilde{A}}^{c}\;\cap \;{\tilde{B}}_{2}}y^{2}dy\right]\\ \; & =\;\;\int_{{\tilde{B}}_{2}}\tilde{h}\cdot y^{2}dy\;\;=\;\;\frac{\tilde{h}}{12}{\left(\mathrm{vol}\left(\tilde{A}\right)\right)}^{3}, \end{array}$$

where in (*a*) we use the interval set ${\tilde{B}}_{2}\;≜\;\left[-\mathrm{vol}\left(\tilde{A}\right)/2,\;\mathrm{vol}\left(\tilde{A}\right)/2\;\right]$, centered around zero, and of the same total length as $\tilde{A}$ with the resulting property that $\mathrm{vol}\left({\tilde{A}}^{c}\cap {\tilde{B}}_{2}\right)=\mathrm{vol}\left(\tilde{A}\cap {\tilde{B}}_{2}^{c}\right)$. So that

(A28) $${\tilde{c}}_{1}{\tilde{h}}^{-1/3}\;\;\geq \;\;\mathrm{vol}\left(\tilde{A}\right),$$

where ${\tilde{c}}_{1}≜{\left(12c_{Y}\right)}^{1/3}$. Continuing (A27), with (A28) we obtain the following upper bound on the second term in (A25), which is by (A26) also an upper bound on both terms of (A25):

(A29) $$\begin{array}{cc} \; & \int_{R}p_{Y}\left(y\right)\log_{b}\;p_{Y}\left(y\right)dy\;-\;\int_{R}p_{Y}^{q}\left(y\right)\log_{b}\;p_{Y}^{q}\left(y\right)dy\;\;\overset{\tilde{h}\;\leq \;1/e}{\leq }\;\;{\tilde{c}}_{1}{\tilde{h}}^{2/3}\max\left\{\log_{b}\;\left(1/\tilde{h}\right),\;\log_{b}\left(e\sqrt{K}\right)\right\}. \end{array}$$

So if $\delta_{1}>0$ in (A9), then the possible decrease caused by substitution of $p_{Y}^{q}$ in place of $p_{Y}$ on the LHS of (86) is at most o(1).
*The LHS of (85)*
Let us define two functions of y∈R:

(A30) $$Q_{\beta }\left(y\right)\;≜\;\Delta_{\beta ,\;n}\cdot \left\lfloor y/\Delta_{\beta ,\;n}+1/2\right\rfloor ,\;\;\;\;\;\;r_{\beta }\left(y\right)\;≜\;y\;-\;Q_{\beta }\left(y\right).$$

Then with $p_{XY}^{q}$ defined in (A6), we can obtain a lower bound for the expression on the LHS of (85):

(A31) $$\begin{array}{l} \begin{array}{ccc} \;\Delta_{\alpha ,n}\Delta_{\beta ,n}\sum_{\begin{array}{c} x\;\in \;X_{n}\\ y\;\in \;Y_{n} \end{array}}p_{XY}^{q}\left(x,y\right){\left(y-x\right)}^{2} & = & \Delta_{\alpha ,n}\sum_{x\;\in \;X_{n}}\int_{R}p_{XY}^{q}\left(x,y\right){\left(y-x-r_{\beta }\left(y\right)\right)}^{2}dy\\ \overset{a}{\leq }\;\;\Delta_{\alpha ,n}\sum_{x\;\in \;X_{n}}\int_{R}p_{XY}\left(x,y\right){\left(y-x\right)}^{2}dy & + & \Delta_{\alpha ,n}\Delta_{\beta ,n}^{2}\sum_{x\;\in \;X_{n}}\int_{R}p_{XY}\left(x,y\right)dy\\ & + & \Delta_{\alpha ,n}\Delta_{\beta ,n}\sum_{x\;\in \;X_{n}}\int_{R}p_{XY}\left(x,y\right)\left|y-x\right|dy \end{array}\\ \overset{b}{\leq }\;\;\sum_{x\;\in \;X_{n}}P_{X}\left(x\right)\int_{R}p_{Y|X}\left(y\;|\;x\right){\left(y-x\right)}^{2}dy\;+\;\Delta_{\beta ,n}^{2}\;+\;\Delta_{\beta ,n}{\left[\sum_{x\;\in \;X_{n}}P_{X}\left(x\right)\int_{R}p_{Y|X}\left(y\;|\;x\right){\left(y-x\right)}^{2}dy\right]}^{1/2}\\ \overset{c}{\leq }\;\;\sum_{x\;\in \;X_{n}}P_{X}\left(x\right)\int_{R}p_{Y|X}\left(y\;|\;x\right){\left(y-x\right)}^{2}dy\;+\;\underset{o(1)}{\underset{︸}{\Delta_{\beta ,\;n}^{2}\;+\;\Delta_{\beta ,\;n}\sqrt{c_{XY}}}}, \end{array}$$

where (*a*) follows because $p_{XY}^{q}\left(x,y\right)\leq p_{XY}\left(x,y\right)$ and $|\;r_{\beta }\left(y\right)\;|\;\leq \;\Delta_{\beta ,\;n}/2$, (*b*) follows by (A3) and Jensen’s inequality for the concave (∩) function $f\left(t\right)=\sqrt{t}$, and (*c*) follows by the condition of the lemma.
*Joint type $P_{XY}$*
Let us define two mutually complementary probability masses for each $\left(x,y\right)\in X_{n}\times Y_{n}$:

(A32) $$\begin{array}{cc} P_{XY}^{q}\left(x,y\right)\;\; & ≜\;\;p_{XY}^{q}\left(x,y\right)\Delta_{\alpha ,n}\Delta_{\beta ,n}, \end{array}$$

(A33) $$\begin{array}{cc} P_{XY}^{a}\left(x,y\right)\;\; & ≜\;\;\{\begin{array}{cc} P_{X}\left(x\right)-\sum_{\tilde{y}\;\in \;Y_{n}}P_{XY}^{q}\left(x,\tilde{y}\right), & \;\;\;y=Q_{\beta }\left(x\right),\\ 0, & \;\;\;o.w., \end{array} \end{array}$$

where $Q_{\beta }\left(\cdot \right)$ is defined in (A30). It follows from (A6) and (8), that each number $P_{XY}^{q}\left(x,y\right)$ is an integer multiple of 1/n and $\sum_{y\;\in \;Y_{n}}P_{XY}^{q}\left(x,y\right)\leq P_{X}\left(x\right)$ for each $x\in X_{n}$. Then a joint type can be formed with the two definitions above:

(A34) $$P_{XY}\left(x,y\right)\;\;≜\;\;P_{XY}^{q}\left(x,y\right)\;+\;P_{XY}^{a}\left(x,y\right),\;\;\;\;\;\;\forall \left(x,y\right)\in X_{n}\times Y_{n},$$

such that $P_{XY}\in P_{n}\left(X_{n}\times Y_{n}\right)$ and $\sum_{y\;\in \;Y_{n}}P_{XY}\left(x,y\right)=P_{X}\left(x\right)$ for each $x\in X_{n}$.
*The RHS of (85)*
Having defined $P_{XY}$ and $P_{XY}^{q}$, let us examine the possible decrease in the expression found on the RHS of (85) when $P_{XY}$ inside that expression is replaced with $P_{XY}^{q}$:

(A35) $$\begin{array}{cc} \; & \sum_{\begin{array}{c} x\;\in \;X_{n}\\ y\;\in \;Y_{n} \end{array}}P_{XY}\left(x,y\right){\left(y-x\right)}^{2}\;-\;\sum_{\begin{array}{c} x\;\in \;X_{n}\\ y\;\in \;Y_{n} \end{array}}P_{XY}^{q}\left(x,y\right){\left(y-x\right)}^{2}\\ \overset{a}{=} & \sum_{\begin{array}{c} x\;\in \;X_{n}\\ y\;\in \;Y_{n} \end{array}}P_{XY}^{a}\left(x,y\right){\left(y-x\right)}^{2}\;\;\overset{b}{=}\;\;\sum_{\begin{array}{c} x\;\in \;X_{n}\\ y\;\in \;Y_{n} \end{array}}P_{XY}^{a}\left(x,y\right)r_{\beta }^{2}\left(x\right)\;\;\overset{c}{\leq }\;\;p_{1}\Delta_{\beta ,n}^{2}/4\;\;\overset{d}{\leq }\;\;\underset{o(1)}{\underset{︸}{c_{1}h^{1/2}\Delta_{\beta ,\;n}^{2}/2}}, \end{array}$$

where (*a*) follows by (A34), (*b*) follows according to the definitions (A30) and (A33), (*c*) follows because $|\;r_{\beta }\left(x\right)\;|\;\leq \;\Delta_{\beta ,\;n}/2$ and because

(A36) $$\begin{array}{cc} \sum_{\begin{array}{c} x\;\in \;X_{n}\\ y\;\in \;Y_{n} \end{array}}P_{XY}^{a}\left(x,y\right)\;\; & \underset{\left(A34\right)}{=}\;\;1\;-\;\sum_{\begin{array}{c} x\;\in \;X_{n}\\ y\;\in \;Y_{n} \end{array}}P_{XY}^{q}\left(x,y\right)\;\;\underset{\left(A32\right)}{=}\;\;1\;-\;\Delta_{\alpha ,n}\Delta_{\beta ,n}\sum_{\begin{array}{c} x\;\in \;X_{n}\\ y\;\in \;Y_{n} \end{array}}p_{XY}^{q}\left(x,y\right)\\ \; & \overset{\left(A6\right)}{=}\;\;1\;-\;\int \int_{R^{2}}p_{XY}^{q}\left(x,y\right)dxdy\;\;\overset{\left(A23\right)}{=}\;\;p_{1}, \end{array}$$

then (*d*) follows by the upper bound on $p_{1}$ of (A23). Since by definition (A32) we also have

$$\sum_{\begin{array}{c} x\;\in \;X_{n}\\ y\;\in \;Y_{n} \end{array}}P_{XY}^{q}\left(x,y\right){\left(y-x\right)}^{2}\;\;=\;\;\Delta_{\alpha ,\;n}\Delta_{\beta ,\;n}\sum_{\begin{array}{c} x\;\in \;X_{n}\\ y\;\in \;Y_{n} \end{array}}p_{XY}^{q}\left(x,y\right){\left(y-x\right)}^{2},$$

which is exactly the beginning of (A31), then combining (A31) and (A35) we obtain (85). The remainder of the proof for (84) and (86) will easily follow by Lemma A1 applied to corresponding discrete entropy expressions with probability masses.
*The RHS of (84)*
In order to upper-bound the expression on the RHS of (84), it is convenient to write:

(A37) $$\begin{array}{cc} \; & \sum_{\begin{array}{c} x\;\in \;X_{n}\\ y\;\in \;Y_{n} \end{array}}P_{XY}\left(x,y\right)\;\log_{b}\;\frac{P_{XY}\left(x,y\right)}{\Delta_{\alpha ,\;n}\Delta_{\beta ,\;n}}\;-\;\sum_{\begin{array}{c} x\;\in \;X_{n}\\ y\;\in \;Y_{n} \end{array}}P_{XY}^{q}\left(x,y\right)\;\log_{b}\;\frac{P_{XY}^{q}\left(x,y\right)}{\Delta_{\alpha ,\;n}\Delta_{\beta ,\;n}}\\ \overset{a}{=}\;\; & \sum_{\begin{array}{c} x\;\in \;X_{n}\\ y\;\in \;Y_{n} \end{array}}\left[\;P_{XY}\left(x,y\right)\;\log_{b}\;P_{XY}\left(x,y\right)\;-\;P_{XY}^{q}\left(x,y\right)\;\log_{b}\;P_{XY}^{q}\left(x,y\right)\;\right]\;+\;\sum_{\begin{array}{c} x\;\in \;X_{n}\\ y\;\in \;Y_{n} \end{array}}P_{XY}^{a}\left(x,y\right)\;\log_{b}\;\frac{1}{\Delta_{\alpha ,\;n}\Delta_{\beta ,\;n}}\\ \overset{b}{\leq }\;\; & \sum_{\begin{array}{c} x\;\in \;X_{n}\\ y\;\in \;Y_{n} \end{array}}\max\left\{-P_{XY}^{a}\left(x,y\right)\;\log_{b}\;P_{XY}^{a}\left(x,y\right),\;\;P_{XY}^{a}\left(x,y\right)\;\log_{b}\;e\right\}\;+\;\left(1-\gamma \right)p_{1}\;\log_{b}\;n\\ \overset{c}{\leq }\;\; & \sum_{\begin{array}{c} x\;\in \;X_{n}\\ y\;\in \;Y_{n} \end{array}}P_{XY}^{a}\left(x,y\right)\;\log_{b}\;n\;+\;\left(1-\gamma \right)p_{1}\;\log_{b}\;n\;\;\overset{d}{=}\;\;\left(2-\gamma \right)p_{1}\;\log_{b}\;n\;\;\overset{e}{\leq }\;\;\underset{o(1)}{\underset{︸}{4c_{1}h^{1/2}\;\log_{b}\;n}}, \end{array}$$

where (*a*) follows by (A34); for (*b*) in the first term we apply the upper bound of Lemma A1 with its parameters $t_{1}=P_{XY}^{q}\left(x,y\right)$ and $t_{1}+t=P_{XY}\left(x,y\right)\leq 1$ with $t=P_{XY}^{a}\left(x,y\right)\leq p_{1}$ by (A34), (A36), for *n* sufficiently large such that $p_{1}\leq 1/e$; while in the second term we use the equality (A36) and (7); (*c*) follows for n>2 since $P_{XY}^{a}\left(x,y\right)\geq 1/n$ when positive; (*d*) and (*e*) follow respectively by the equality (A36) and the inequality (A23). Now since

$$\sum_{\begin{array}{c} x\;\in \;X_{n}\\ y\;\in \;Y_{n} \end{array}}P_{XY}^{q}\left(x,y\right)\;\log_{b}\;\frac{P_{XY}^{q}\left(x,y\right)}{\Delta_{\alpha ,\;n}\Delta_{\beta ,\;n}}\;\;=\;\;\int \int_{R^{2}}p_{XY}^{q}\left(x,y\right)\log_{b}\;p_{XY}^{q}\left(x,y\right)dxdy,$$

the inequality in (84) follows by comparing (A10), (A22), and (A37).
*The RHS of (86)*
With $P_{Y}^{q}\left(y\right)≜\sum_{x\;\in \;X_{n}}P_{XY}^{q}\left(x,y\right)$ and $P_{Y}^{a}\left(y\right)≜\sum_{x\;\in \;X_{n}}P_{XY}^{a}\left(x,y\right)$ we have

$$\begin{array}{cc} \; & \sum_{y\;\in \;Y_{n}}P_{Y}\left(y\right)\;\log_{b}\;\frac{P_{Y}\left(y\right)}{\Delta_{\beta ,\;n}}\;-\;\sum_{y\;\in \;Y_{n}}P_{Y}^{q}\left(y\right)\;\log_{b}\;\frac{P_{Y}^{q}\left(y\right)}{\Delta_{\beta ,\;n}}\;\;= \end{array}$$

(A38) $$\begin{array}{cc} \overset{a}{=}\;\; & \sum_{y\;\in \;Y_{n}}\left[\;P_{Y}\left(y\right)\;\log_{b}\;P_{Y}\left(y\right)\;-\;P_{Y}^{q}\left(y\right)\;\log_{b}\;P_{Y}^{q}\left(y\right)\;\right]\;+\;\sum_{y\;\in \;Y_{n}}P_{Y}^{a}\left(y\right)\;\log_{b}\;\frac{1}{\Delta_{\beta ,\;n}}\\ \overset{b}{\geq }\;\; & \sum_{y\;\in \;Y_{n}}P_{Y}^{a}\left(y\right)\;\log_{b}\;P_{Y}^{a}\left(y\right)\;+\;\beta p_{1}\;\log_{b}\;n\\ \overset{c}{\geq }\;\; & \sum_{y\;\in \;Y_{n}}P_{Y}^{a}\left(y\right)\;\log_{b}\;\left(1/n\right)\;+\;\beta p_{1}\;\log_{b}\;n\;\;\overset{d}{=}\;\;-\left(1-\beta \right)p_{1}\;\log_{b}\;n\;\;\overset{e}{\geq }\;\;\underset{o(1)}{\underset{︸}{-2c_{1}h^{1/2}\;\log_{b}\;n}}, \end{array}$$

where (*a*) follows by (A34); for (*b*) in the first term, we apply the lower bound of Lemma A1 with its parameters $t_{1}=P_{Y}^{q}\left(y\right)$ and $t_{1}+t=P_{Y}\left(y\right)$ with $t=P_{Y}^{a}\left(y\right)\leq p_{1}$ by (A34) and (A36), for *n* sufficiently large such that $p_{1}\leq 1/e$, while in the second term, we use the equality (A36) and (7); (*c*) follows because $P_{Y}^{a}\left(y\right)\geq 1/n$ whenever positive, (*d*) and (*e*) follow, respectively, by the equality (A36) and the inequality (A23). From (A8) and (A32) we observe that $P_{Y}^{q}\left(y\right)=p_{Y}^{q}\left(y\right)\Delta_{\beta ,n}$. Since the function $p_{Y}^{q}\left(y\right)$ is piecewise constant in R by the definition of $p_{XY}^{q}$, it follows that

$$\sum_{y\;\in \;Y_{n}}P_{Y}^{q}\left(y\right)\;\log_{b}\;\frac{P_{Y}^{q}\left(y\right)}{\Delta_{\beta ,n}}\;\;=\;\;\int_{R}p_{Y}^{q}\left(y\right)\log_{b}\;p_{Y}^{q}\left(y\right)dy.$$

Then the inequality (86) follows by comparing (A29), (A38). This concludes the proof of Lemma 18. □

**Lemma** **A1.**
*Let f(x)=xlnx, then for 0<t≤1/e and $t_{1}>0\;$,*

$$t\ln t\;\;\leq \;\;f\left(t_{1}+t\right)-f\left(t_{1}\right)\;\;\leq \;\;t\;\ln\max\left\{1/t,\;(t_{1}+t)e\right\}.$$

**Proof.** For t≤1/e, the magnitude of the derivative of f(x) in the interval (0,t) is monotonically decreasing and its average is −lnt, while in the adjacent interval [t,1/(et)], the magnitude of the derivative is upper-bounded by −lnt: $\;\;\;|\;f^{\prime }\left(x\right)\;|\;\leq \;-\ln t,\;\;\;t\leq x\leq 1/\left(et\right)$. Then there are three possible cases: (1) $\;t_{1}\;<\;t\;<\;t_{1}+t\;<\;1/\left(et\right)\;$:

$$\begin{array}{cc} |\;f\left(t_{1}+t\right)-f\left(t_{1}\right)\;|\;\; & \leq \;\;|\;f\left(t_{1}+t\right)-f\left(t\right)\;|\;+\;|\;f\left(t_{1}\right)-f\left(t\right)\;|\\ \; & =\;\;|\;f\left(t_{1}+t\right)-f\left(t\right)\;|\;+\;t_{1}\ln t_{1}\;-\;t\ln t\\ \; & \leq \;\;-t_{1}\ln t\;+\;t_{1}\ln t_{1}\;-\;t\ln t\\ \; & \leq \;\;-t\ln t. \end{array}$$

(2) $\;t\;\leq \;t_{1}\;<\;t_{1}+t\;\leq \;1/\left(et\right)\;$:

$$\begin{array}{c} |\;f\left(t_{1}+t\right)-f\left(t_{1}\right)\;|\;\;\leq \;\;-t\ln t. \end{array}$$

(3) $\;1/\left(et\right)\;<\;t_{1}+t\;$: $\;\;\;\;\;\;\;\;\;\;\;\;\;\;\;\;\;\;\;\;\;\;\;\;\;\;\;\;\;\;0\;\;\leq \;\;f\left(t_{1}+t\right)-f\left(t_{1}\right)\;\;\leq \;\;f^{\prime }\left(t_{1}+t\right)\;t\;\;=\;\;\left(\ln(t_{1}+t)+1\right)\;t$. □
